# The prevalence of chaotic dynamics in games with many players

**DOI:** 10.1038/s41598-018-22013-5

**Published:** 2018-03-20

**Authors:** James B. T. Sanders, J. Doyne Farmer, Tobias Galla

**Affiliations:** 1 Theoretical Physics, School of Physics and Astronomy, The University of Manchester, Manchester, M13 9PL United Kingdom; 2 Institute for New Economic Thinking at the Oxford Martin School, University of Oxford, Oxford, OX2 6ED UK; 3 Mathematical Institute, University of Oxford, Oxford, OX1 3LP UK; 4 Department of Computer Science, University of Oxford, Oxford, OX1 3QD UK; 5 Santa Fe Institute, Santa Fe, NM 87501 USA

## Abstract

We study adaptive learning in a typical p-player game. The payoffs of the games are randomly generated and then held fixed. The strategies of the players evolve through time as the players learn. The trajectories in the strategy space display a range of qualitatively different behaviours, with attractors that include unique fixed points, multiple fixed points, limit cycles and chaos. In the limit where the game is complicated, in the sense that the players can take many possible actions, we use a generating-functional approach to establish the parameter range in which learning dynamics converge to a stable fixed point. The size of this region goes to zero as the number of players goes to infinity, suggesting that complex non-equilibrium behaviour, exemplified by chaos, is the norm for complicated games with many players.

## Introduction

Many branches of science are interested in systems made up of a large number of competing individuals. Examples range from financial markets and other social systems, to populations undergoing biological evolution, to networked computer systems. In many such situations individuals compete for limited resources, and the natural model is a game, which consists of a set of players who at any point in time choose from a set of possible actions in an attempt to maximize their payoff. Game theory has received a great deal of attention since the mid-20th century^1^, but research has overwhelmingly focused on simple games, with only a very small number of players and pure strategies, even though real game-like systems often involve large numbers of individuals and possible strategies. While many of the observed properties of simple games carry over directly to more complicated ones, it is becoming increasingly clear that complicated games can show important types of behaviour not found in simpler systems.

Early work in game theory focused on the concept of equilibria, in particular the famous *Nash equilibrium*^2^, in which the players adopt strategies such that no player can improve her own payoff by unilaterally changing her own strategy. The strategies at a Nash equilibrium can be probabilistic combinations of pure strategies, called *mixed strategies*. Nash’s ideas have been particularly influential in economics, where agents are often assumed to adopt Nash equilibria. It should be emphasized that this is a behavioural assumption, and that the empirical evidence for this is mixed^3^.

Equilibrium models are perfectly plausible in the context of simple games when there is a unique Nash equilibrium that is easy to calculate. In complicated games, there are typically numerous distinct Nash equilibria^4,5^, and locating even one of them can be a laborious task: the computing time of the best known algorithms increases exponentially with the size of the game^6^. This seems to cast doubt on whether it is reasonable to assume that players of complicated games naturally discover equilibria. But if they don’t play equilibria, what do they do instead? And is there a way to determine *a priori* whether players will converge to a unique Nash equilibrium using a reasonable learning strategy?

Opper and Diederich studied replicator dynamics, a standard model of biological evolution, in the context of complicated games. They found that the dynamics converges to a unique fixed point in some regions of parameter space, but that in other regions, the dynamics does not settle down^7,8^. Sato and co-workers showed that adaptation learning can result in chaotic dynamics even in low-dimensional games^9–11^. Building on this earlier work, Galla and Farmer studied complicated two-player games in which the strategies are randomly generated but fixed in time, assuming that players use an experience-weighted attraction dynamics to learn their strategies^12^. They found that there are distinct regions of the parameter space with different behaviours. When the timescale for learning is short and the payoffs of the players are strongly negatively correlated, they observed convergence to unique fixed points. But when the time scale for learning is long and when the payoffs are less negatively correlated they observed limit cycles and chaos. And when the timescale for learning is long and the payoffs are positively correlated they observed a large multiplicity of stable fixed points.

In this paper we extend this analysis to *p*-player games and find that for large numbers of players complex dynamics is not merely frequent but ubiquitous. The region of parameter space in which the players’ strategies consistently converge to a unique fixed point appears to vanish as *p* → ∞. This suggests that complex non-equilibrium behaviour may be the norm in dynamics on complicated games with many players, at least under the type of learning we study here.

Our goal can be understood through an analogy to fluid flow. It is well known that fluid flow can be characterized *a priori* in terms of a few key parameters that can be estimated on the back of an envelope. The most famous of these is the Reynolds number, which is a non-dimensional ratio of stress and viscosity. Though the precise transition point depends on other parameters, if the Reynolds number is high then the flow is likely to be turbulent. Our goal here is similar: We seek parameters that can characterize *a priori* whether or not a game will exhibit complex dynamics in the strategy space as the players learn. Here we are particularly interested in what happens as the number of players increases. Since the presence of many players makes the game more complex, we hypothesize that it will tend to make the strategy dynamics more complex as well. (And indeed this is what we observe).

The remainder of the paper is organized as follows: We introduce the experience-weighted attraction learning algorithm and define what we mean by a complex *p*-player game. We then present an overview of the different types of behaviour seen in the learning of such games, along with a more quantitive analysis based on numerical simulations. We then turn to a semi-analytical study of *p*-player learning based on a generating-functional approach. This technique allows us to derive estimates for the boundaries of stable and complex behaviour in parameter space for games with a large number of strategies. Finally, we present a brief discussion of volatility clustering and the relevance of our result for the modeling of financial markets before we summarize our results. The Supplement contains further details of the numerical methods used to identify the different types of dynamical behaviour, and of the generating functional analysis. It also contains some additional numerical results.

## Model

### Experience-weighted attraction

Suppose that a set of players repeatedly play a game in which they each choose from a distinct set of strategies without conferring with each other. The players have good memories and a full understanding of the payoffs that a given combination of strategies would yield for them and are only interested in maximizing their own payoffs. We are interested in the case where the players learn their strategies based on past experience. A common approach assumes the players adapt behaviour over time based on the past success of each possible strategy^13–16^. The basic idea is that the players calculate a numerical score, known as an ‘attraction’ or ‘propensity’, for each possible strategy, describing how successful they expect it to be. They then select a strategy based on the relative score of each possible action, play the game one or more times, then use the outcome to update their attractions for future play. This defines an adaptation process in which agents learn from past experience and continuously try to improve their actions.

Two types of simple learning models have proved especially popular over the years. In reinforcement learning, the players calculate the attraction of a strategy based on how successful it has been when they have employed it in the past. In belief-based learning, the attractions are determined according to how successful the possible strategies would have been, had they been used in prior iterations. The experience-weighted attraction (EWA) system introduced by Camerer and Ho^13^ combines these two approaches into a single algorithm–in fact, belief-based learning can be seen as a deterministic limit of reinforcement learning in which the players sample all pure strategies at each time step. Combined with a logit model of how the players choose strategies based on their attractions, EWA is observed to be a reasonably good match for how people learn to play simple games^14,15^.

We are interested in how often EWA converges to equilibria, so we select the deterministic (belief-based) version of the model. While the noisy (reinforcement) version may well perform stochastic oscillations about equilibria in the right conditions, we would expect that in general, the introduction of noise would lead to complex dynamics being observed even more often. Both cases were studied by Galla and Farmer and the differences were not dramatic (see the Supplementary Material of reference^12^).

Consider a *p*-player game, where each player has *N* actions to choose from. The rewards for the players are defined by the generalised payoff matrix ${{\rm{\Pi }}}_{i,{i}_{\mu +1},\ldots ,{i}_{\mu -1}}^{\mu }$, which represents the payoff to player *μ* if she plays action *i*, while the other players *μ* + 1, …, *μ* − 1 play actions *i*_*μ*+1_, …, *i*_*μ*−1_, respectively (where the subscripts labelling the players are to be interpreted modulo *p*).

We use update rules similar to those of reference^12^ but adapted to the multi-player game,

1 $$\begin{array}{rcl}{x}_{i}^{\mu }(t+\mathrm{1)} & = & \frac{\exp [\beta {Q}_{i}^{\mu }(t)]}{\sum _{k}\exp [\beta {Q}_{k}^{\mu }(t)]},\\ {Q}_{i}^{\mu }(t+\mathrm{1)} & = & \mathrm{(1}-\alpha ){Q}_{i}^{\mu }(t)+\sum _{\,{i}_{\mu +1},\ldots ,{i}_{\mu -1}}{{\rm{\Pi }}}_{i,{i}_{\mu +1},\ldots ,{i}_{\mu -1}}^{\mu }\prod _{\kappa \ne \mu }{x}_{{i}_{\kappa }}^{\kappa }(t\mathrm{)}.\end{array}$$

Here **x** represents the players’ strategies, with ${x}_{i}^{\mu }(t)$ denoting the probability that player *μ* will choose action *i* at time *t*. The value ${Q}_{i}^{\mu }(t)$ is player *μ*’s *attraction* to action *i* at time *t*. The two parameters of the system are the memory loss rate *α*, which lies in the interval [0, 1], and the *intensity of choice β*, which is non-negative. A player’s attraction to an action is essentially a geometrically discounted average of the payoffs that would have been achieved by playing that action in earlier time steps, with a discount factor determined by *α*. The intensity of choice *β* determines the bias with which players choose actions with higher attractions–if *β* = 0, then the players ignore the attractions and choose each action with equal probability, while in the limit as *β* → ∞, the players each choose their most attractive action with probability 1. The intensity of choice therefore plays a similar role to the inverse temperature in thermodynamics.

Our system is identical to that studied by Galla and Farmer^12^, except that they restricted the number of players to be *p* = 2. Our contribution is to understand how this changes as *p* becomes larger. The system of Camerer and Ho^14,15^, as well as allowing for noise, allows the intensity of choice *β* to vary over time. In the present work we assume that it has attained its long-term value. Thus the dynamics we study here are a special case of^14,15^.

The attractions ${Q}_{{i}}^{\mu }$ can be eliminated from the update rules in Eq. (1) to yield

2 $${x}_{i}^{\mu }(t+\mathrm{1)}=\frac{1}{{Z}^{\mu }(t)}{x}_{i}^{\mu }{(t)}^{1-\alpha }\exp (\beta \sum _{{i}_{\mu +1},\ldots ,{i}_{\mu -1}}{{\rm{\Pi }}}_{i,{i}_{\mu +1},\ldots ,{i}_{\mu -1}}^{\mu }\prod _{\kappa \ne \mu }{x}_{{i}_{\kappa }}^{\kappa }(t)),$$

where

3 $${Z}^{\mu }(t)=\sum _{k}{x}_{k}^{\mu }{(t)}^{1-\alpha }\exp (\beta \sum _{{i}_{\mu +1},\ldots ,{i}_{\mu -1}}{{\rm{\Pi }}}_{k,{i}_{\mu +1},\ldots ,{i}_{\mu -1}}^{\mu }\prod _{\kappa \ne \mu }{x}_{{i}_{\kappa }}^{\kappa }(t))$$

is a normalization factor.

### Constructing typical complicated games with *p* players

We are interested in the behaviour of the EWA system for generic complicated games. To that end, we draw the payoff matrix **Π** from a multivariate normal distribution with

4 $$\langle {{\rm{\Pi }}}_{{i}_{\mu },{i}_{\mu +1},\ldots ,{i}_{\mu -1}}^{\mu }{{\rm{\Pi }}}_{{i}_{\nu },{i}_{\nu +1},\ldots ,{i}_{\nu -1}}^{\nu }\rangle =(\begin{array}{cl}1 & \mu =\nu \\ \frac{{\rm{\Gamma }}}{p-1} & \mu \ne \nu \end{array}$$

and all other correlations zero. The multivariate Gaussian distribution is a natural choice because it is the maximum entropy distribution when there are constraints on the first and second moments. We have chosen the construction above because it yields the only possible multivariate Gaussian distribution that satisfies the following properties: (i) the distribution is symmetric with respect to the different players and pure strategies (that is, swapping any two players or pure strategies would leave the distribution unchanged) and (ii) the payoffs for any two distinct choices of actions are uncorrelated (by choice of actions we mean the tuple (*i*_1_, …, *i*_*p*_) representing the pure strategies played by the *p* players). As before, we wish to emphasize that once Π is chosen it remains fixed through the duration of the iterated game.

The parameter Γ can be seen as the level of ‘zero-sumness’ of the game, and must lie on the interval [−1, *p* −1]. When Γ = *p* − 1, any outcome of the game leads to each player receiving the same payoff with probability 1. When Γ = 0, all payoffs are uncorrelated. When Γ = −1, for any given outcome, the players’ payoffs sum to zero with probability 1. Therefore Γ can be seen as a ‘competition parameter’–at large positive values, the players have common goals, while at large negative values, they are working against each other. When *p* = 2, the possible values of Γ span the range −1 ≤ Γ ≤ 1. However when *p* > 2 the situation becomes more complicated. Eq. (4) indicates that the payoffs each have variance unity, the correlations between payoffs for different outcomes are uncorrelated, and the correlation between the payoffs of two different players for any given outcome is Γ.

#### Rescaling the intensity of choice

The intensity of choice parameter *β* plays an important role and must be handled with care. As already mentioned, in the limit as *β* → 0 all choices become equally likely and learning is trivial–the learning algorithm automatically converges to a fixed point. For the learning to be sensible *β* must be large enough so that successful past moves are favored over unsuccessful moves. As explained in detail in the Supplement, in the large *N* limit the variable ${x}_{i}^{\mu }$ tends to get smaller as 1/*N* and the expected payoff from different actions scales as $1/\sqrt{{N}^{p-1}}.$ This means that in the limit as *N* → ∞ the intensity of choice automatically needs to be higher in order to compensate for these two effects. We therefore write *β* as

5 $$\beta =\tilde{\beta }\sqrt{{N}^{p-1}}.$$

As explained in the Supplement, this is equivalent to rescaling ${x}_{i}^{\mu }$ so that ${\sum }_{i}{x}_{i}^{\mu }=N$ and rescaling the payoffs so that their standard deviation scales as $1/\sqrt{{N}^{p-1}}.$ This is also more convenient for our analytical treatment. *Thus from here on we replace β according to* Eq. (5), *and to simplify the notation we do not write the twiddle*.

Following^12^, we focus on the continuous-time limit of the EWA system. Letting

$$r=\beta /\alpha ,$$

this limit is found by keeping *r* fixed while taking *α* → 0 and *β* → 0 simultaneously, and rescaling time. It is important to note that both the geometric discounting of the past profits of an action (i.e., the memory-loss) and the logit selection remain significant in this limit. The strength of memory loss is parametrised by *α*, and the intensity of choice by *β*. The ratio *α*/*β* therefore represents the relative strength of the two contributions. Sending *α* and *β* to zero does not mean that we eliminate either of these effects, instead we keep the relative strength of the effects (the ratio *α*/*β*) constant when we take the continuous-time limit. This yields the so-called Sato-Crutchfield dynamics^9,10^

6 $$\frac{{\dot{x}}_{i}^{\mu }(t)}{{x}_{i}^{\mu }(t)}=-\frac{1}{r}\,\mathrm{ln}\,{x}_{i}^{\mu }(t)+\sum _{{i}_{\mu +1},\ldots ,{i}_{\mu -1}}{{\rm{\Pi }}}_{i,{i}_{\mu +1},\ldots ,{i}_{\mu -1}}^{\mu }\prod _{\kappa \ne \mu }{x}_{{i}_{\kappa }}^{\kappa }(t)-{\rho }^{\mu }(t),$$

where *ρ*^*μ*^(*t*) = *lnZ*^*μ*^(*t*)/*β*. A detailed derivation can be found for example in the Supplementary Material of ref.^12^. For small values of *r* memory loss dominates the dynamics while for large values of *r* memory loss is weak. In both cases this is measured relative to the intensity of choice. Note that, since each player satisfies the constraint that the probability of taking any given action sums to one, the resulting dynamical system for the learning dynamics is of dimension (*N* − 1)*p*.

We assume throughout this work that *α* and *β* are small enough that the continuous limit is a good approximation. We take this limit mainly for analytical convenience; the continuous limit is easier to study, as it has only one relevant parameter, the ratio of *α* to *β*.

### Strategy for exploring the parameter space

For a *p*-player game the payoff “matrices” each have *p* indices, and so are not two dimensional matrices in the normal usage of the world, but are *p*-dimensional. This significantly complicates the problem of exploring the parameter space numerically. For a game in which a single player can take one of *N* actions the payoff matrix for a single player has *N*^*p*^ components; for *p* players there are *pN*^*p*^ components. For *p* = 10 and *N* = 10, for example, this means that 10^11^ (a hundred billion) random numbers must be generated in order to construct the game. The sheer amount of memory needed for simulation creates a serious bottleneck.

Given the numerical constraints this forces us to rely more heavily on the analytic calculation than Galla and Farmer did when they explored two-player games. We use numerical simulations to get a feeling for the behaviour of the system, with relatively small values of *N* and *p*. In parallel we perform an analytic calculation of the stability boundary between the region where there is unique convergence to a fixed point and the rest of the parameter space. We then compare the analytic and numerical simulations and demonstrate that the analytic calculation seems to be reasonably accurate, given the magnitude of the finite-size effects. Finally we use the analytic calculation to assess the behaviour in the limit where *N* → ∞ and *p* is large.

## Numerical exploration of the parameter space

### Overview

In this section we give an overview of our numerical exploration of the parameter space. As observed by Galla and Farmer, the strategies of the players can converge to any of the possible types of attractors, including fixed points, limit cycles and chaos. In some regimes a given game may have multiple fixed points, i.e. multiple basins of attraction, but we have not observed this when the attractors are limit cycles or chaos. In Fig. l we show some examples. The chaotic behaviour can be low dimensional, as shown in the middle row, or high dimensional, as shown in the last row. For high-dimensional chaos a given action typically has epochs in which it is almost never selected and others in which it is used frequently–the range of variation is striking.

**Figure 1:**
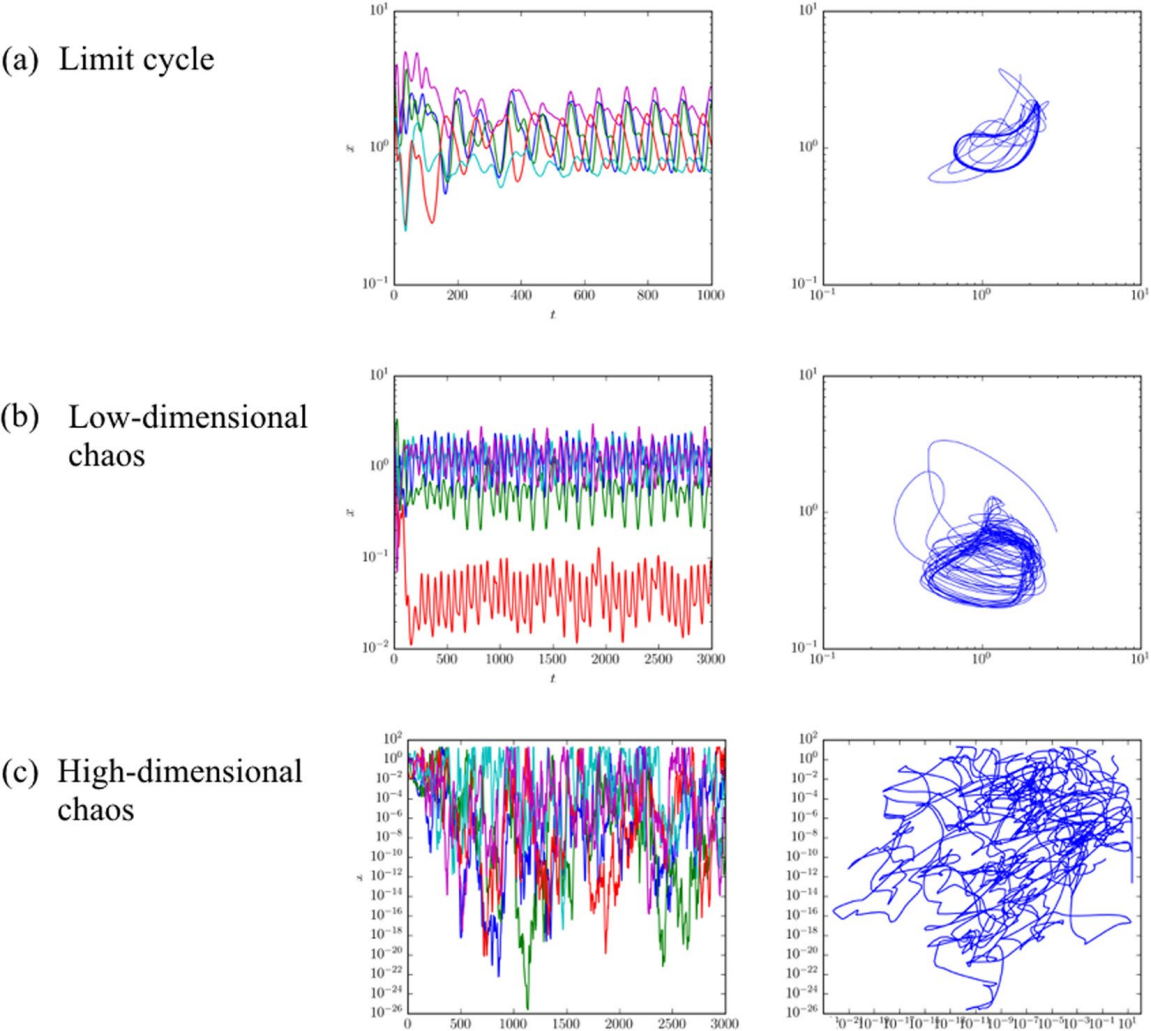
Time series and phase plots showing complex dynamics under EWA learning, including (**a**) limit cycle, (**b**) low-dimensional chaos, and (**c**) high-dimensional chaos. The game has three players (*p* = 3) and *N* = 20 possible actions, with *β* = 0.05 and Γ = −0.5. The time series plots on the left show the probability ${x}_{i}^{\mu }$ for player *μ* to use action *i* as a function of time for five different actions, and the phase plots on the right shows the probability for two of the actions as a function of each other. Case (a) illustrates that limit cycles can have complicated geometric forms and long periods. For smaller values of *α*/*β* and negative Γ, chaos is very common, ranging from low-dimensional chaotic attractors as shown in (b) to high-dimensional attractors as shown in (c). Note that for high dimensional chaos the probability that a given action is used at different points in time can vary by as much as a factor of 10^20^. Memory-loss parameter in the different panels is (a) *α* = 0.038, (b) *α* = 0.037, and (c) *α* = 0.01.

**Figure 2:**
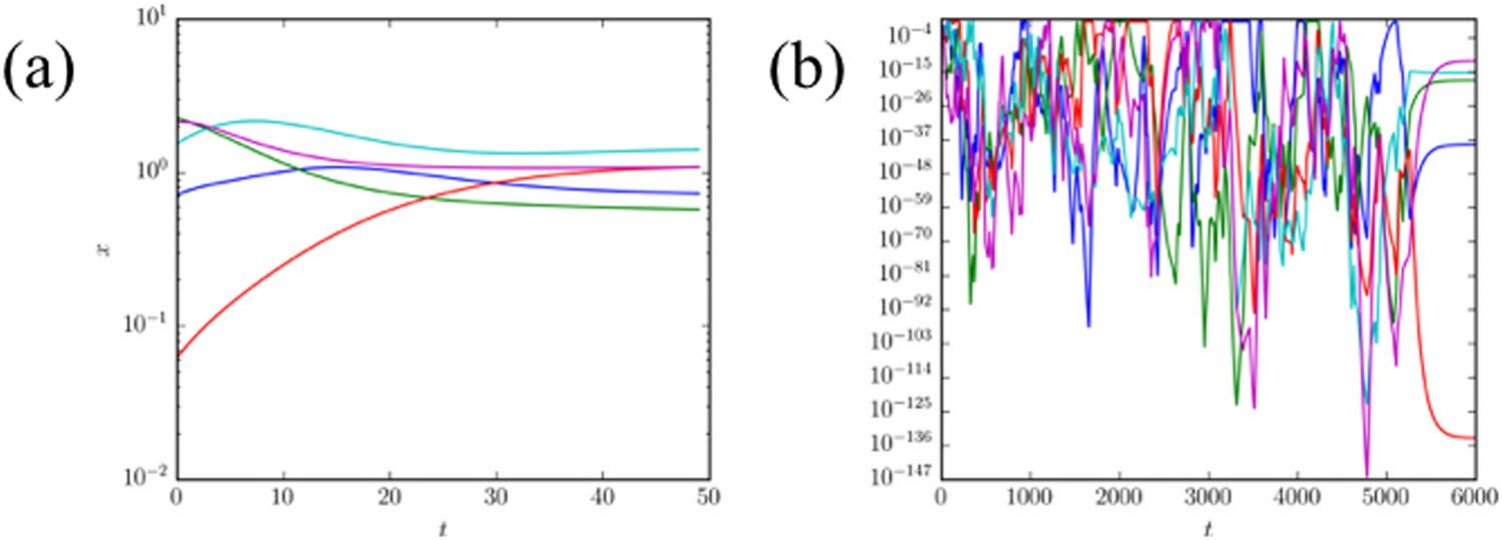
Trajectories for EWA system leading to a fixed point in a three-player game. Panel (a) shows an instance in which a fixed point is reached relatively quickly. Panel (b) illustrates a metastable chaotic transient eventually collapsing to a fixed point. In both examples each player has a choice of *N* = 20 possible actions and the intensity of choice is *β* = 0.05. A random sample of five of the players’ strategy components ${x}_{i}^{\mu }$ are plotted. Remaining parameters are *α* = 0.1, Γ = −0.5 in panel (a), and *α* = 0.01, Γ = 0.1 in panel (b).

When the system converges to a fixed point, it usually does so rather quickly, as shown in Fig. 2(a). However there are sometimes long metastable chaotic-looking transients that suddenly collapse to a fixed point, as shown in Fig. 2(b). This is particularly likely for small values of *α*/*β* and small positive Γ (i.e., for weakly positively correlated payoffs and players with long memories).

Broadly speaking the dynamics of the system is governed by two elements: the payoff matrices and memory loss. In absence of memory loss the players’ propensities are accumulated over the entire history of play. This can drive the system towards chaos, limit cycles or multiple fixed points, depending on the correlation of payoff matrix elements. If there are multiple attractors, initial conditions determine which one is reached. The memory-loss term adds a damping force directed towards a fully mixed strategy in the interior of phase space. Varying the ratio *α*/*β* controls the relative strength of both contributions. For small *α*/*β* memory loss is weak and chaos, limit cycles or multiple fixed points can then be seen. If memory loss is large enough initial conditions are eventually forgotten and a unique fixed point is reached for all starting points.

**Figure 3:**
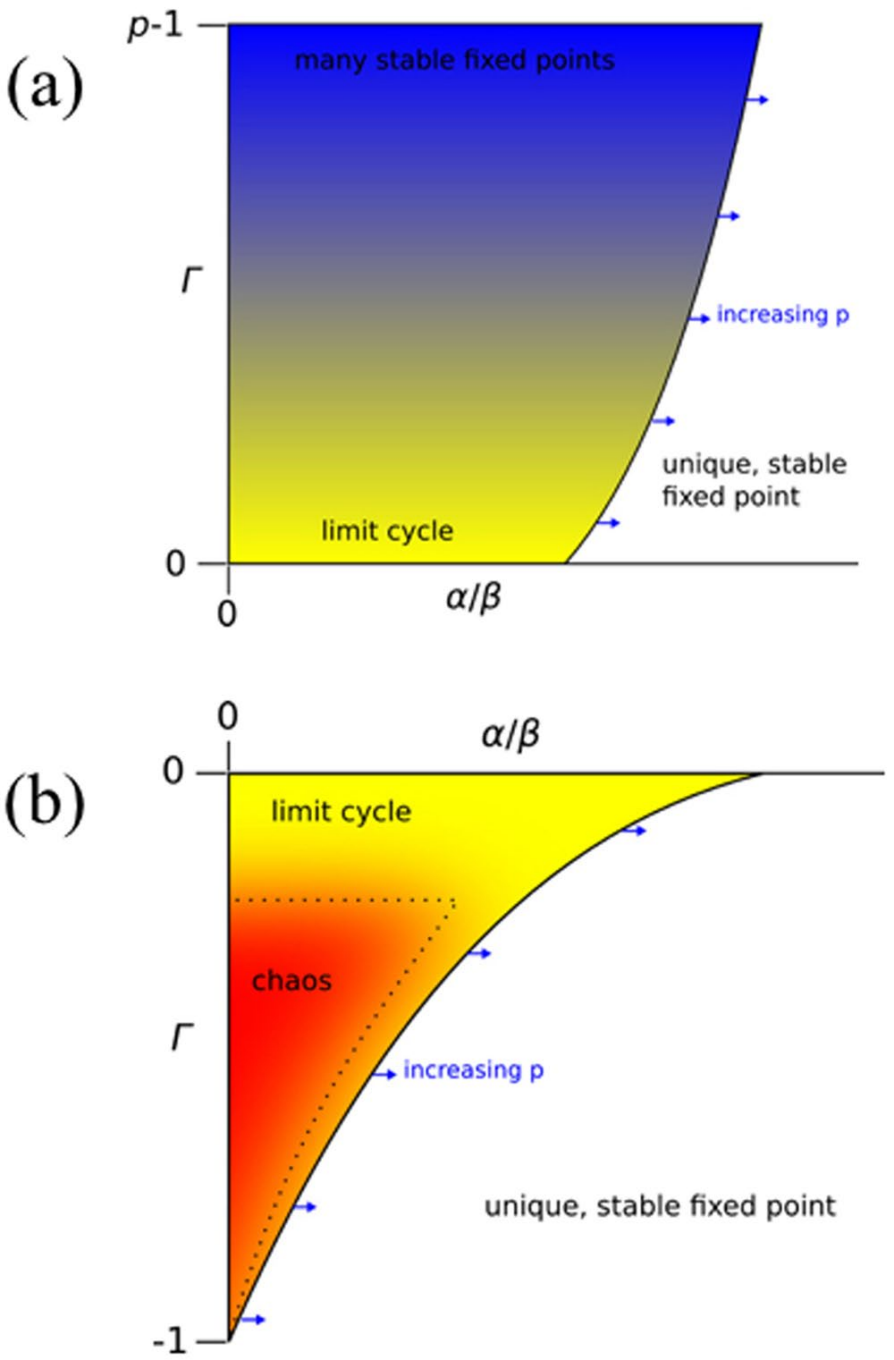
Schematic phase diagrams describing the observed long-term behaviour of the *p*-player EWA system for large but finite *N*. In (**a**) Γ > 0, meaning players’ payoffs are positively correlated. Here we observe a unique stable equilibrium for large *α*/*β* and multiple stable equilibria for small *α*/*β*. In (**b**) Γ < 0, meaning players’ payoffs are anti-correlated. Here we once again observe a unique stable equilibrium for large *α*/*β*, but we now observe chaos for small *α*/*β*. Limit cycles are common near the boundaries, particularly near Γ ≈ 0. The solid line in panel (b) illustrates that there is a region in which the dynamics is either chaotic or runs into a limit cycle. The dotted line indicates that, within that unstable region, there is a smaller region of particularly high dimensional chaos. This is for illustration only, quantitative details of the phase boundaries between stable and unstable dynamics will be discussed below.

Although the boundaries can be a bit fuzzy, the parameter space divides into distinct regions. These are illustrated in the schematic diagram in Fig. 3. We briefly describe the main features:

#### Positively correlated payoffs

For Γ > 0 and large *α*/*β* the dynamics converge to a unique fixed point. Holding Γ constant, for small *α*/*β* the dynamics converges to one of multiple fixed points, see also Fig. S3 in the Supplement. Which fixed point is reached depends on the initial conditions for the deterministic dynamics.

#### Negatively correlated payoffs

For Γ < 0 and large *α*/*β* the dynamics converge to a unique fixed point (just as for Γ > 0). Holding Γ constant, for small *α*/*β* the dynamics are chaotic. This corresponds to longer memory. As Γ increases from Γ = −1 to Γ = 0 the size of the chaotic region increases. The stability boundary dividing the region with complex dynamics from the unique equilibrium shifts to the right (i.e. toward a larger value of *α*/*β*) as the number of players increases.

#### Uncorrelated payoffs

Limit cycles are common near the boundaries, particularly near the boundary where Γ ≈ 0, see also Figs S1 and S2 in the Supplement. In general the behaviours reported here are not strict, in the sense that generating different payoff matrices with the same values of *α*/*β* can result in different behaviours, particularly near the boundaries. We conjecture that these are finite size effects, so that the boundaries would become distinct and the behaviour at a given set of parameters would become crisp in the limit as *N* → ∞.

#### Prevalence of chaotic dynamics

The key result is that as the number of players increases the size of the region with complex dynamics grows. If Γ > 0, this means that the region with multiple fixed points becomes larger; if Γ < 0 this means that the region with chaotic dynamics becomes larger. More players make the system less likely to converge to a unique equilibrium. This is particularly important for zero-sum game (Γ = −1); in this case for *p* = 2 chaos is only observed in the limit as *α*/*β* → 0, whereas for *p* > 2 chaos is observed over a finite interval in *α*/*β*.

As already mentioned, it is not possible to perform numerical experiments for large values of both *p* and *N*. We will present some numerical evidence for these results, but they make more sense when guided by the theory.

## Generating functional approach

We now turn to treat the learning dynamics analytically. In the language of the theory of disordered system the random payoff matrix in our problem represents quenched disorder. Techniques from spin glass physics can be applied to study the thermodynamic limit (i.e, the limit of large payoff matrices, *N* → ∞). We use the Martin-Siggia-Rose generating functional to derive an effective dynamics. For a recent review of these methods see^17^.

Broadly speaking the calculation proceeds as follows: in a first step the average over the disorder (the random payoff matrices) is carried out and the effective dynamics is derived. This process is subject to coloured noise, and reflects the statistics over all games within the Gaussian ensemble. In a second step, a fixed point of this dynamics is assumed. We investigate the linear stability of this fixed point, and calculate the boundary to the phase in parameter space where more complex dynamics are seen. Thus we cannot calculate where the dynamics follow limit cycles or chaos, but we can calculate the boundary between the unique stable fixed point and other behaviours. As discussed later, we can only do this for Γ < 0. We note that similar calculations have been carried out for replicator dynamics on two-player and in multi-player games^7,18^, see also^19^ for approaches to *p*-player random games using static replica methods.

### Effective process

The first step is to set up a generating functional to describe the probability measure of all possible paths of the dynamics. Performing the average over the assignment of payoff matrices an ‘effective dynamics’ can then be derived. This calculation is based on path integrals, and somewhat lengthy. We do not report it here in the main paper, instead details are relegated to the Supplement, Section S1. The outcome of the calculation is a stochastic integro-differential equation for the evolution of the distribution of components *x*(*t*) of the players’ strategies in the large-*N* limit. This process is of the form

7 $$\frac{\dot{x}(t)}{x(t)}={\rm{\Gamma }}\int {\rm{d}}t^{\prime} G(t,t^{\prime} )C{(t,t^{\prime} )}^{p-2}x(t^{\prime} )-\frac{1}{r}\,\mathrm{ln}\,x(t)-\rho (t)+\eta (t),$$

where *η*(*t*) is a colored Gaussian random variable satisfying 〈*η*(*t*)*η*(*t*′)〉_*_ = *C*(*t*, *t*′)^*p*−1^ and 〈*η*(*t*)〉_*_ = 1. We use 〈⋅〉_*_ to denote an average over realizations of the effective dynamics. The dynamical order parameters *C*(*t*, *t*′) and *G*(*t*, *t*′) are correlation and response functions of the learning dynamics. They are determined from

8 $$G(t,t^{\prime} )={\langle \frac{\delta x(t)}{\delta \eta (t^{\prime} )}\rangle }_{\ast },\quad C(t,t^{\prime} )={\langle x(t)x(t^{\prime} )\rangle }_{\ast }.$$

The effective process in Eq. (7) together with Eq. (8) define a self-consistent system for *C* and *G*. The function *ρ*(*t*) in the effective process is a Lagrange multiplier ensuring normalization. It is defined such that 〈*x*〉_*_ = 1. Note that in the derivation of the effective dynamics, it is assumed that each component of each player’s strategy is initially drawn from an identical distribution.

### Fixed point solution

We now focus on the dynamics at large values of *α*/*β*. Numerical simulations of the learning dynamics suggest that one unique stable fixed point is found for any one realization of the game in this regime. We therefore make a fixed point ansatz for the effective dynamics. In such a stationary fixed point regime the response function *G*(*t*, *t*′) becomes a function of the time difference only, i.e., *G*(*t*, *t*′) = *G*(*t* − *t*′), while the correlation function tends to a constant, *C*(*t*, *t*′) ≡ *q*, see also^7,12,18^ for further details. Within the fixed-point ansatz the random variable *η*(*t*) in Eq. (7) tends to a constant value drawn from a Gaussian distribution with zero mean and variance *q*^*p*−1^. Fixed points of the effective dynamics are then found from

9 $${x}^{\ast }({\rm{\Gamma }}{q}^{p-2}\chi {x}^{\ast }-\frac{1}{r}\,\mathrm{ln}{x}^{\ast }+{\eta }^{\ast }-{\rho }^{\ast })=0,$$

where $q={\langle {(x\ast )}^{2}\rangle }_{\ast }$ and $\chi ={\int }_{0}^{\infty }{\rm{d}}\tau G(\tau )$, and x*, η*, and ρ* are the fixed point values of *x*, *η*, and *ρ*, respectively. We can write η^*^ = *q*^(*p*−1)/2^*z*, where *z* is a standard Gaussian random variable. Then, dropping the stars, we have

10 $$x(z)({\rm{\Gamma }}{q}^{p-2}\chi x(z)-\frac{1}{r}\,\mathrm{ln}\,x(z)+{q}^{\frac{p-1}{2}}z-\rho )=0,$$

where *χ*, *q*, and *ρ* are to be determined from

11 $$\begin{array}{rcl}{\langle \frac{\partial x(z)}{\partial z}\rangle }_{\ast } & = & {q}^{\frac{p-1}{2}}\chi ,\\ {\langle x{(z)}^{2}\rangle }_{\ast } & = & q,\\ {\langle x(z)\rangle }_{\ast } & = & 1.\end{array}$$

These relations can be re-written as

12 $$\begin{array}{rcl}{\int }_{-\infty }^{\infty }{\rm{D}}z\frac{\partial x(z)}{\partial (z)} & = & {q}^{\frac{p-1}{2}}\chi ,\\ {{\int }_{-\infty }^{\infty }{\rm{D}}zx(z)}^{2} & = & q,\\ {\int }_{-\infty }^{\infty }{\rm{D}}zx(z) & = & \mathrm{1,}\end{array}$$

where $${\rm{D}}z=\frac{{\rm{d}}z}{\sqrt{2\pi }}{e}^{-{z}^{2}\mathrm{/2}}$$.

The relation in Eq. (10) can be re-arranged to give an explicit expression for *x*(*z*) in terms of the so-called Lambert W function *W*( · ). The value *W*(*y*) is defined as the solution of the equation *We*^*W*^ = *y*. Restricting *W* and *y* to the real line, the solution exists for *y* ≥ −1/*e*. It is uniquely defined for *y* ≥ 0 and double valued for −1/*e* < *y* < 0. We find

13 $$x=-\frac{1}{{\rm{\Gamma }}r{q}^{p-2}\chi }W(-{\rm{\Gamma }}r{q}^{p-2}\chi {e}^{r({q}^{(p-1)/2}z-\rho )}).$$

We note that it is not clear that Eq. (13) has valid solutions for all choices of the model parameters. If these do not exist the fixed point ansatz is invalid, and so we do not expect the dynamics to settle down. There may also be instances in which Eq. (10) has multiple solutions for *x* for a given value of the standard Gaussian variable *z*. In principle the distribution of fixed points could be composed of any mixture of these solutions. If the argument of the Lambert function is positive however, there is a unique and well defined solution, *x*(*z*), for any value of *z*. Throughout this discussion it is important to keep in mind that the macroscopic order parameters *q*, *χ* and *ρ* are to be determined self-consistently via Eq. (12).

### Stability analysis

By numerically solving the fixed point equations, we see that for a given value of *p*, stable fixed points exist for large values of 1/*r* but not for small values. We now proceed to determine the boundary of stability. Suppose the effective process in Eq. (7) is perturbed from a fixed point by a small noise term *ξ*(*t*). We then have

14 $$\dot{x}(t)=x(t)[{\rm{\Gamma }}\int {\rm{d}}t^{\prime} G(t,t^{\prime} )C{(t,t^{\prime} )}^{p-2}x(t^{\prime} )-\frac{1}{r}\,\mathrm{ln}\,x(t)-\rho (t)+\eta (t)+\xi (t)].$$

We assume that *ξ*(*t*) is white Gaussian noise of unit amplitude. Writing $x(t)={x}^{\ast }+\hat{x}(t)$, and $\eta (t)={\eta }^{\ast }+\hat{\eta }(t)$, and keeping only linear terms in *ξ*, $\hat{x}$, and $\hat{\eta }$, we obtain

15 $$\hat{x}(t)=-\frac{1}{r}\hat{x}(t)+{x}^{\ast }[{\rm{\Gamma }}\int {\rm{d}}t^{\prime} G(t-t^{\prime} )C{(t-t^{\prime} )}^{p-2}\mathop{x}\limits^{\frown {}}(t^{\prime} )+\hat{\eta }(t)+\xi (t)].$$

Defining *H*(*t*, *t*′) = *G*(*t*, *t*′)*C*(*t*, *t*′)^*p*−2^, and taking the Fourier transform of Eq. (15), yields

16 $$(\frac{{\rm{i}}\omega +{r}^{-1}}{{x}^{\ast }}-{\rm{\Gamma }}\tilde{{H}}(\omega ))\tilde{x}(\omega )=\tilde{\eta }(\omega )+\tilde{\xi }(\omega ),$$

where the tildes denote Fourier transforms. This leads to the relation

17 $${\langle {|\tilde{x}(\omega )|}^{2}\rangle }_{\ast }=((p-\mathrm{1)}{\langle {({x}^{\ast })}^{2}\rangle }_{\ast }^{p-2}{\langle {|\tilde{x}(\omega )|}^{2}\rangle }_{\ast }+{\langle {|\tilde{\xi }(\omega )|}^{2}\rangle }_{\ast })\times {\langle {|A(\omega ,{x}^{\ast })|}^{-2}\rangle }_{\ast },$$

where

18 $${\mathscr{A}}(\omega ,{x}^{\ast })=\frac{{\rm{i}}\omega +{r}^{-1}}{{x}^{\ast }}-{\rm{\Gamma }}\tilde{H}(\omega \mathrm{)}.$$

We can write this as

19 $${\langle {|\tilde{x}(\omega )|}^{2}\rangle }_{\ast }={({\langle {|{\mathscr{A}}(\omega ,{x}^{\ast })|}^{-2}\rangle }_{\ast }^{-1}-(p-\mathrm{1)}{q}^{p-2})}^{-1}.$$

The left-hand side is positive by definition so the calculation runs into a contradiction if the expression on the right-hand side turns negative. As it approaches zero (from above) the magnitude of fluctuations diverges. The fixed point can only be stable when

20 $${\langle {|{\mathscr{A}}(\omega ,{x}^{\ast })|}^{-2}\rangle }_{\ast } < \frac{1}{(p-\mathrm{1)}{\langle {(x\ast )}^{2}\rangle }_{\ast }^{p-2}}.$$

In order to identify the onset of instability we follow the procedure detailed in Reference^7^ and focus on *ω* = 0. We obtain

21 $${\langle {|\frac{1}{r{x}^{\ast }}-{\rm{\Gamma }}{q}^{p-2}\chi |}^{-2}\rangle }_{\ast } < \frac{1}{(p-\mathrm{1)}{q}^{p-2}},$$

i.e.,

22 $${\int }_{-\infty }^{\infty }{\rm{D}}z{(\frac{1}{rx(z)}-{\rm{\Gamma }}{q}^{p-2}\chi )}^{-2}\le \frac{1}{(p-\mathrm{1)}{q}^{p-2}}.$$

For negative values of Γ, the position of the stability boundary can be determined straightforwardly by numerically solving Eqs (12, 13) for a given set of model parameters, and by subsequently evaluating the stability condition Eq. (22) throughout parameter space. As already mentioned, this procedure fails for Γ > 0.

### The large-*p* limit

In general the location of the stability region defined by (22) cannot be determined fully analytically. However, it is possible to make progress in several limits as shown below and make a good sketch of the behaviour when Γ < 0.

Taking Γ → 0 in Eq. (10) we find *x*(*z*) = exp[*r*(*q*^(*p*−1)/2^*z* − *ρ*)], and using Eq. (12) the order parameters *r*, *q*, and *ρ* satisfy

23 $$\begin{array}{rcl}\chi  & = & r,\\ \exp ({r}^{2}{q}^{p-1}) & = & q,\\ \rho  & = & \frac{\mathrm{ln}\,q}{2r},\end{array}$$

in this limit. The second expression is equivalent to

24 $$q={(-\frac{W-(p-1){r}^{2}}{(p-1){r}^{2}})}^{1/(p-1)},$$

where *W*( · ) is the Lambert W function. For (*p* − 1)*r*^2^ > 1/*e* this has no solutions. For (*p* − 1)*r*^2^ < 1/*e* it has two, given by the two branches of the Lambert W function. The solution corresponding to the upper branch (*W* > −1) satisfies (22) so is always stable, while the other is always unstable. Therefore, on the Γ = 0 line, there is a stable fixed point only for large values of 1/*r*, with the stability boundary given by

25 $$\begin{array}{rcl}r=\chi  & = & \frac{1}{\sqrt{(p-1){\rm{e}}}},\\ q & = & \exp (\frac{1}{p-1}),\\ \rho  & = & \frac{1}{2}\sqrt{\frac{e}{p-1}}.\end{array}$$

For Γ = −1 and *p* = 2 the system is stable as soon as there is non-zero memory loss (*α* > 0), that is to say the stability line passes through the point (1/*r* = 0, Γ = −1) when *p* = 2, see also^12^. For larger numbers of players we find that the stability boundary never reaches the 1/*r* = 0 line.

**Figure 4:**
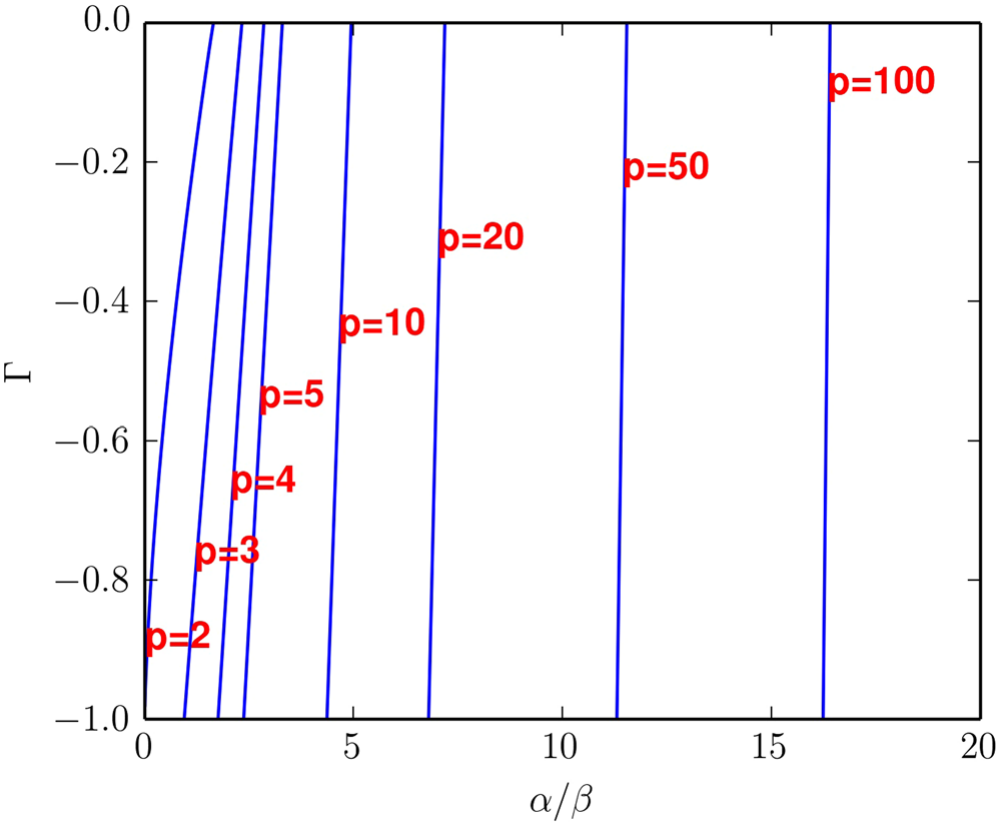
Stability boundaries of the effective dynamics for several values of *p* as a function of *α*/*β* and Γ, for the case where Γ < 0. Each curve is the stability boundary for the stated value of *p*. To the left of any curve the fixed point of the effective dynamics is unstable, to the right it is stable. The key result is that the stability boundary moves to the left as *p* increases, so the size of the regime with complex dynamics grows.

The boundary between the two regions crosses the Γ = 0 line at the location we have determined analytically, and tends to a straight line as *p* → ∞, as shown in Fig. 4. It is in fact possible to demonstrate this analytically, see the Supplement (Section S2) for details.

**Figure 5:**
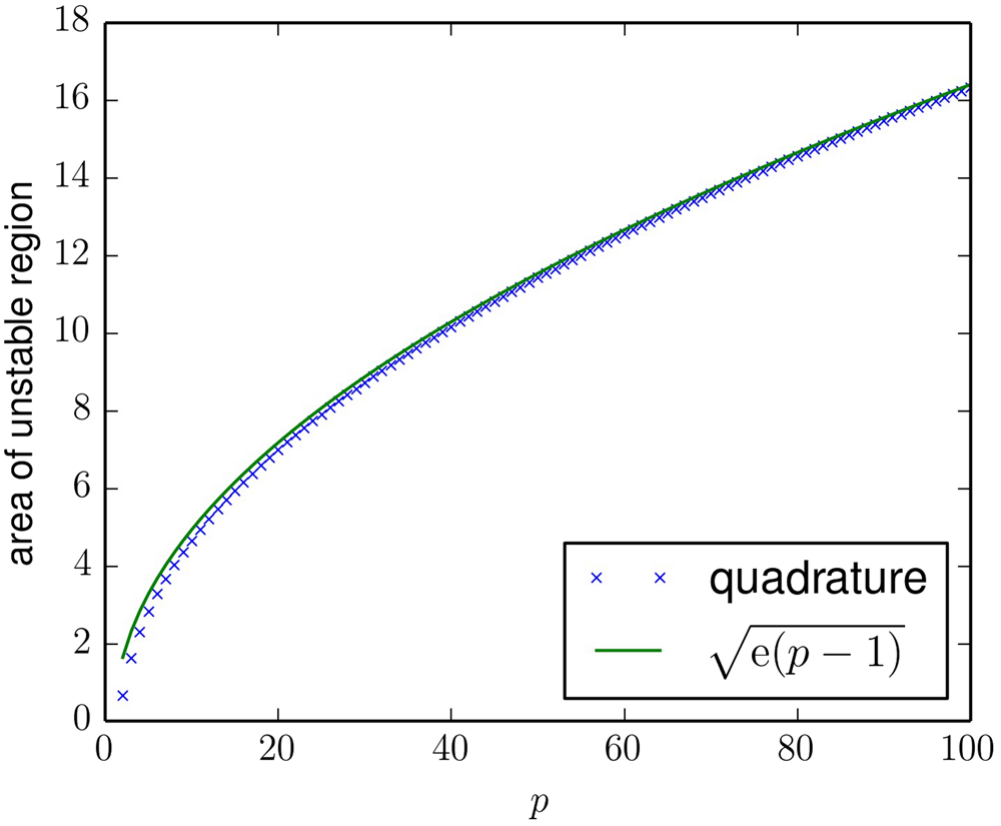
Plot showing the area of the unstable region for negative Γ as a function of the number of players, *p*. This area is estimated numerically using Gaussian quadrature on results obtained for *β* = 0.01; this is valid in the limit as *N* → ∞. The analytic estimate of the area is $\sqrt{{\rm{e}}(p-1)}$, see Eq. (26). This indicates that the area of the parameter space with complex dynamics goes to infinity proportional to $\sqrt{p}$ as *p* → ∞.

Based on this result we can estimate that the size, *A*, of the unstable region in the *α*/*β* − Γ plane in Fig. 4 (restricted to −1 ≤ Γ ≤ 0). The horizontal axis in Fig. 4 shows *α*/*β* = 1/*r*, the dynamics is chaotic (unstable) on the left, and stable to the right of the corresponding stability lines. For large *p* the line separating unstable and stable dynamics is essentially vertical, located at *α*/*β* = 1/*r*, with *r* given as in the first expression of Eq. (25) i.e., $1/r=\sqrt{(p-1)e}$. As the number of players *p* increases this area grows as

26 $$A\approx \sqrt{{\rm{e}}(p-1)}$$

as shown in Fig. 5. This is an approximate result, valid for large *p*.

Thus, in the case where Γ < 0 and *p* → ∞, the unstable region takes over the entire parameter space. That is, in situations where the players’ payoffs are anticorrelated, their behaviour will always be complex and will never settle down on an equilibrium.

## Heuristic classification of the dynamic behaviour

It is not necessarily straightforward to classify the long-term behaviour of even low-dimensional dynamical systems using empirical data from numerical simulations. For high-dimensional systems such as the EWA dynamics for games with large number of players and/or strategies this task can be extremely challenging.

We experience two major difficulties. Firstly, in some regions of parameter space, transient behaviour can last for a very long time, and can appear chaotic for all intents and purposes even though the system eventually reaches a stable fixed point. Secondly, characterising chaos using Lyapunov exponents or measures of dimension can be problematic for such large systems, for example if the Jacobians are ill conditioned. However, these difficult cases are not the norm, and we can use heuristics to classify behaviour as convergence to a stable fixed point, limit cycle or chaos with a high degree of accuracy.

For a given set of parameters and initial conditions, we iterate the EWA system for a maximum of 500,000 time steps, split into batches of 10,000 steps. After each batch, we explicitly check for the appearance of fixed points or limit cycles. If the relative difference between the maximum and minimum values of each strategy component was less than 1%, we assume a stable fixed point has been found. If there is a *τ* such that all of ${x}_{i}^{\mu }(t+\tau )$, ${x}_{i}^{\mu }(t+2\tau )$, etc. (where *t* was the time at the start of the batch) have components within 0.1% of the components of ${x}_{i}^{\mu }(t)$, then we assume a stable limit cycle has been found. Otherwise, we continue to the next batch. If convergence has not been detected after 500,000 time steps, we assume the system is chaotic.

**Figure 6:**
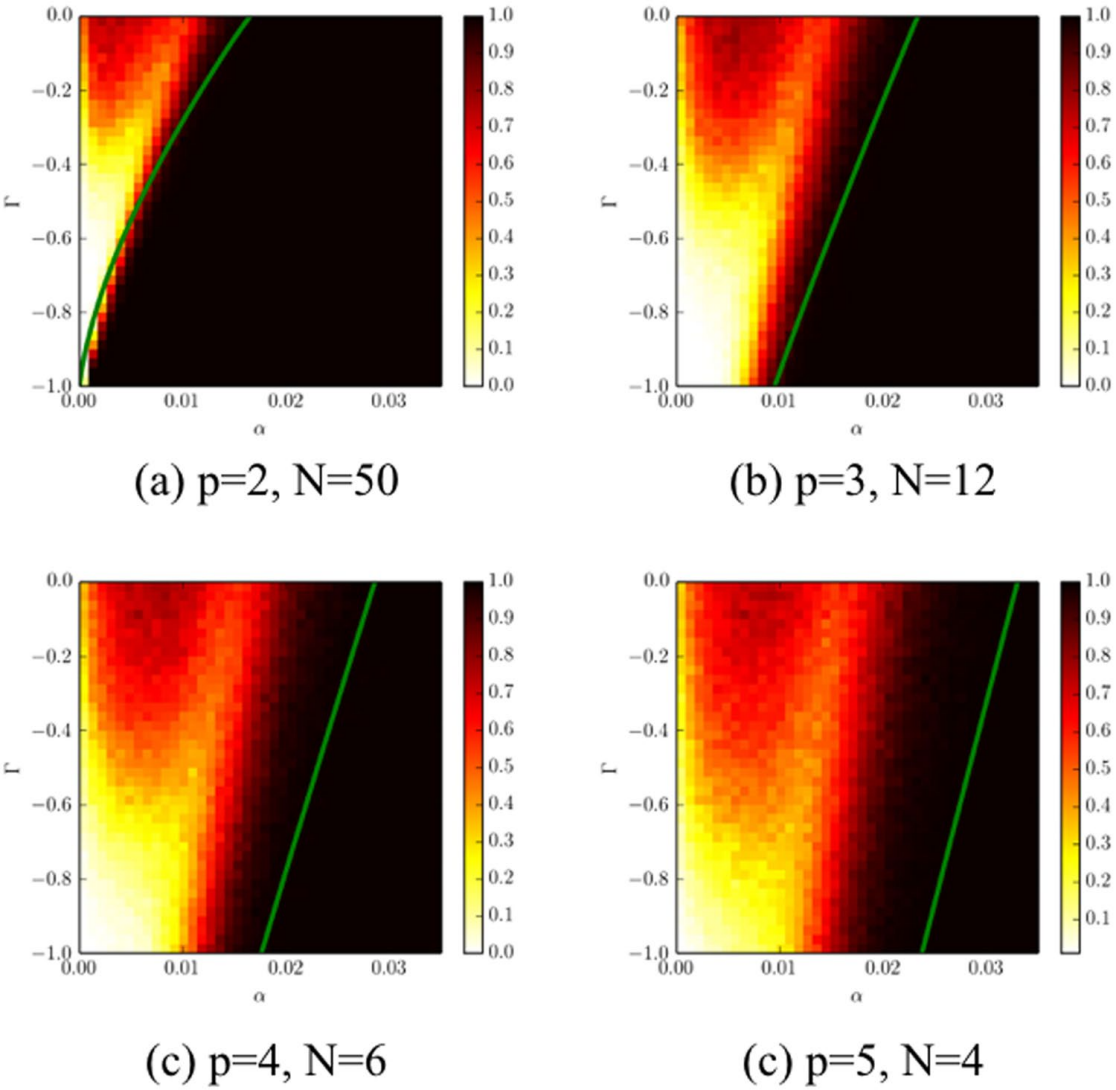
Probability of convergence to a fixed point as a function of the memory parameter *α* and the competition parameter Γ. For each set of parameters we iterate the system from 500 random initial conditions. The heat maps show the fraction that converged to a fixed point. Black means 100% convergence, red (grey) indicates the majority converge, yellow a minority, and white no convergence. The unstable region extends to larger values of *α* as the number of players is increased. The solid curves are derived from the generating functional analysis described above (in the limit *N* → ∞). They separate the region in which a unique stable fixed point is to be expected in this limit (to the right of the green curves) from regions in parameters space where the behaviour is more complex.

This heuristic was used to produce the plots in Fig. 6 and the figures the Supplement.

## Comparison between theory and numerical experiments

We now compare the numerical results to the theoretical predictions. We measure the stability boundary in the numerical experiments by determining whether or not the system converges to a unique fixed point, independent of initial conditions. To do this we choose a set of parameters and initial conditions, iterate the dynamics for a large number of time steps and apply heuristics to check whether the players’ strategies have converged to a fixed point. If we find a fixed point we repeat this for many different initial conditions and check to see whether we always find the same fixed point. We perform a similar procedure to check for limit cycles.

Figure 6 shows how the likelihood of converging to a unique stable fixed point varies throughout the negative-Γ region of parameter space for different values of *N* and *p*. The values we choose are roughly at the limit of what was computationally feasible. We investigate *p* = 2, 3, 4, 5, which constrains the corresponding values of *N* to be *N* = 50, 12, 6, 4. We then sweep Γ and *α* with *β* = 0.05 and construct a heat map showing the likelihood of convergence to a unique stable fixed point. We then compare this to the stability line predicted by the generating functional approach described in the previous section.

The heat map of Fig. 6 is constructed so that black corresponds to convergence to a stable fixed point 100% of the time, red (grey) to convergence roughly 50%–70%, yellow (light grey) 10%–35%, and white to the case in which unique stable fixed points are never found. The behaviour is consistent with what we described schematically in Fig. 3(b): Unique stable fixed points are more likely for higher *α* (i.e. short memory) and the size of the stable region grows with increasing Γ. The region in which complex dynamics are observed grows as *p* increases; in particular for the zero-sum case where Γ = −1 the size of the interval corresponding to complex dynamics is finite and growing with *p*.

The correspondence between the predicted vs. the observed stability boundary gets better as *N* increases. For *p* = 2, where we can make *N* = 50, the correspondence is quite good (Fig. 6(a)); for *p* = 5, where we are only able to make *N* = 4, the correspondence is not as good (Fig. 6(d)); the stability line scales more or less tracks the numerically-observed boundary, but is consistently to the right of it. Given that $N=4\ll N=\infty$, it is not surprising that the approximation is not perfectly accurate. We hypothesize that this is due to finite size effects. To test this, in Fig. S4 in the Supplement we hold the number of players constant at *p* = 2 and systematically vary *N*. We find the correspondence between theory and experiment improving with increasing *N*. In addition the behaviour becomes crisper in the sense that the transition from certain convergence to a unique fixed point to never converging to a unique fixed point happens more suddenly when *N* becomes large. This indicates that the generating function methods gives reasonably good predictions for large *N*, lending confidence to its reliability in the limit as *N* → ∞.

## Chaos and volatility clustering

Before concluding we would like to make a few notes about chaos and volatility clustering. We have so far asserted that much of the behaviour in the competitive region where Γ < 0 is chaotic, without presenting any evidence. In fact we have done extensive computation of Lyapunov exponents using the procedures described in Galla and Farmer^12^. While we experience some numerical problems we can nonetheless state with confidence that the preponderance of the complex dynamics to the right of the stability line corresponds to chaos. Problems arise because it can sometimes be difficult to numerically distinguish chaos and limit cycles without making very long simulations, and because of the metastable chaos observed in Fig. 2, which means that in any given simulation there is a small but nonzero probability that the simulation will eventually collapse to a fixed point. Nonetheless, most of the time we observe chaos, and as *p* increases it tends to be of higher dimension. To prove our main point here and compare to the theory we only needed to determine whether or not we observe convergence to a unique fixed point, which is much easier computationally, so we have chosen not to present evidence based on Lyapunov exponents.

**Figure 7:**
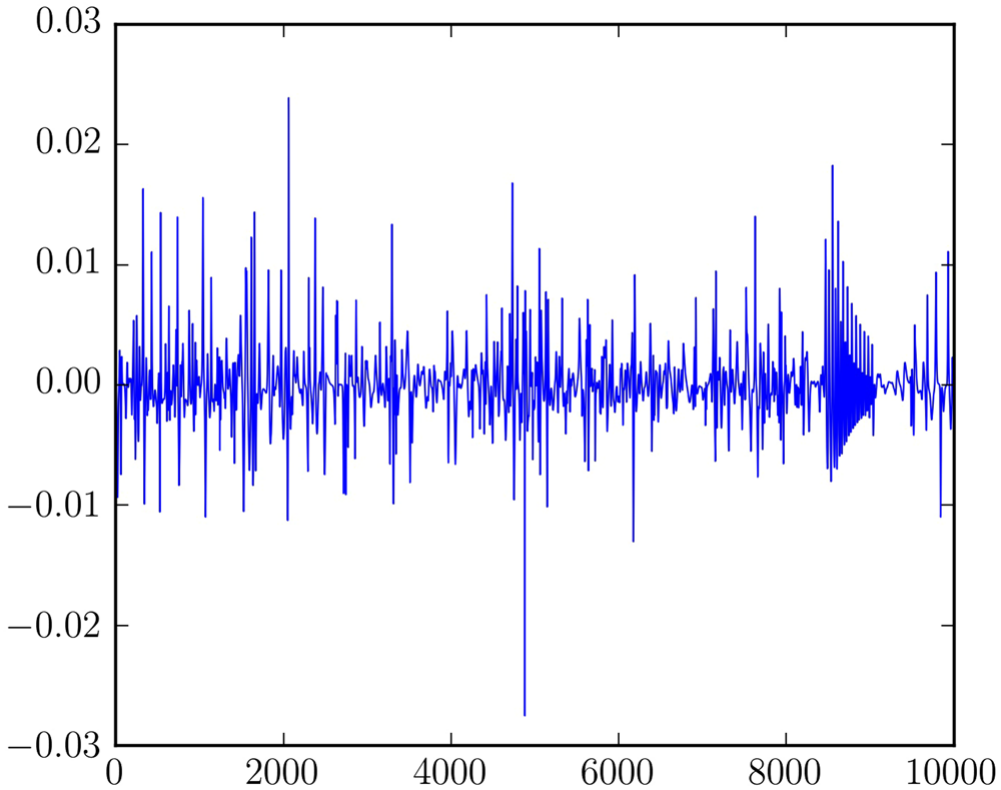
Time series of the changes in the sum of the players’ payoffs for a game with three-players. This corresponds to high dimensional chaos and clustered volatility. By this we mean the tendency for time variability to be positively autocorrelated, with periods of relative calm and periods of relative variability.

In the chaotic regime we consistently observe clustered volatility, similar to that reported for *p* = 2 by Galla and Farmer. By this we mean that the fluctuation in payoffs to the players fluctuates in time in a way that is “clustered”, i.e. positively autocorrelated. There are epochs in which the payoffs are relatively steady and other epochs in which they are highly variable, as shown in Fig. 7. Such intermittency is a common feature of chaotic systems; the dynamics irregularly alternates between qualitatively different types of behaviour. In our system this is seen as a modulation of the amplitude of the high-frequency motion, for example of the variation in payoff. This modulation occurs at a longer timescale and appears to be chaotic, in the sense that there is no obvious periodicity. This is observed both here and in our previous study of 2-player games^12^. For *p* > 2 the chaos tends to be higher dimensional and the clustered volatility stronger. Clustered volatility is common in many real-world situations, including financial time series^20,21^; our work here suggests that this may be a generic result for games in which players learn their strategies using procedures similar to EWA. We conjecture that this is connected to the tendency for a given action to vary from being used frequently for long periods of time to being almost never used, as observed in Fig. l(c), but this remains to be investigated.

## Conclusions

In summary we have characterized the outcome of adaptive learning in complex multi-player games with Gaussian random payoff matrices. The learning dynamics we have simulated is a special case of experience-weighted attraction learning used in behavioural economics. We see different types of dynamical behaviour: convergence to fixed points, limit cycles and chaotic trajectories. Broadly speaking, convergence to a unique stable equilibrium is observed when players’ learning neglects outcomes from the distant past, i.e. when they forget quickly, corresponding to large values of *α*/*β*. In contrast, when they have a long memory, i.e. small *α*/*β*, we observed more complex dynamics. The nature of this dynamics depends on how competitive the game is. For competitive games (i.e. with Γ < 0) the complex dynamics exhibits itself as a limit cycle or chaotic attractor. For cooperative games (i.e. with Γ > 0) the complex dynamics exhibits itself as a multiplicity of fixed points. The boundaries between these behaviours become sharper as *N* increases.

The main focus of this paper was to study the properties of games with more than two players. For two players we replicate the results of Galla and Farmer^12^. For more than two players we are not able to simulate situations with a very large number of actions due to computational constraints, e.g. for *p* = 3 players the largest number of actions we simulated was *N* = 12. To clearly understand the behaviour for large *p* and large *N* we rely on an analytic treatment, which allows us to estimate the stability boundary in the limit as *N* → ∞. Based on tools from the theory of disordered systems, we have carried out a generating-functional analysis of the continuous-time limit of EWA learning, and we have derived approximate semi-analytical results for the onset of stability for games with an infinite number of strategies and with an arbitrary finite number of players, *p*. These results reveal that the parameter range in which learning cannot be expected to settle down to fixed points increases as the number of players in the game grows. This is summarized in Figs 4 and 5. In contrast to the Galla and Farmer paper, where the analytic results were just making the numerical results more rigorous, here the analytic methods were essential to understand the behaviour for large *p*.

In the introduction we posed our objective as seeking a “Reynolds number” for estimating the *a priori* likelihood of complex dynamics, in much the same way that the Reynolds number characterizes turbulence in fluid flow. Indeed the parameter *r* = *β*/*α* characterizing the timescale of the learning process does a reasonably good job in this context: As *r* gets bigger, complex dynamics becomes more likely. The transition also depends on the competitiveness of the game, characterized by Γ, as well as the number of players. Our key result here is that complex dynamics become more likely as the number of players increases. This is not surprising given that games with more players are more complicated, and perhaps also more complex, in the sense that there are more factors to take into account and more inherent degrees of freedom.

The standard theoretical approach in economics assumes convergence to an equilibrium from the outset. Our results here suggest that under circumstances where the players have a long memory of the past, this approach may be inherently flawed. This is particularly true when there are many agents. Our results suggest that there may be large regimes in which the assumption of a unique equilibrium is completely invalid, and where approaches that can accommodate chaotic dynamics, such as agent-based modeling, are needed. Of course in this paper we have only studied one family of learning algorithms, and we have focused on games with many actions. More work is needed to give a definitive answer to the question above. (Since completing this work a by Pangallo *et al*.^22^ has studied six different algorithms and reached conclusions that agree with ours here. They have also studied the behaviour as *N* varies and shown that chaos becomes more likely with increasing *N*).

## Electronic supplementary material

Supplementary Information
