# Supplementary material for: The prevalence of chaotic dynamics in games with many players
Source: Sci Rep. 2018 Mar 20;8:4902. doi: 10.1038/s41598-018-22013-5 (PMC5861132; doi:10.1038/s41598-018-22013-5)
Supplement: Supplementary file 1 — Supplementary Information [file 41598_2018_22013_MOESM1_ESM.pdf]

The prevalence of chaotic dynamics in games with many players
Supplemental Material

James BT Sanders,[1] Tobias Galla,[1] and J. Doyne Farmer[2,3,4]

[1] *Theoretical Physics, School of Physics and Astronomy,*
The University of Manchester, Manchester M13 9PL, UK
[2] *Institute for New Economic Thinking at the Oxford Martin School, University of Oxford, Oxford OX2 6ED, UK*
[3] *Mathematical Institute, University of Oxford, Oxford OX1 3LP, UK*
[4] *Santa Fe Institute, Santa Fe, NM 87501, UK*

S1. RE-SCALING OF VARIABLES

This dynamics in Eq. (1) of the main paper applies to general p-player games, no matter whether the number of actions, N, is finite or infinite. In this notation, the variables x_i^μ denote the probability with which player μ plays action i, and so $\sum_{i=1}^N x_i^\mu = 1$, for all μ. This indicates that each x_i^μ is of order $1/N$.

The term $\sum_{i_{\mu+1},\dots,i_{\mu-1}} \Pi^\mu_{i,i_{\mu+1},\dots,i_{\mu-1}} \prod_{\kappa\neq\mu} x^\kappa_{i_\kappa}$ on the right-hand-side of the second equation in (1) describes the payoff player μ should expect if she plays action i, and given the mixed strategies of her $p-1$ opponents. It is sum over the payoff received for all possible actions of the $p-1$ other players (recall that subscripts labelling players are to be interpreted modulo p). In total, these are N^{p-1} terms.

Given that the payoff matrices elements are drawn at random, each term in the sum on the right-hand-side of Eq. (1) in the main paper is a random variable. So long as all actions are played with non-zero probability, each of the variables $x^\kappa_{i_\kappa}$ can be assumed to be of order $1/N$; their sum is $\sum_{i=1}^N x_i^\mu = 1$, as discussed above. The $\Pi^\mu_{i,i_{\mu+1},\dots,i_{\mu-1}}$ are quenched random variables; they each have a mean of zero, and their variance does not depend on N [see Eq. (4) in the main manuscript]. This turns the term $\sum_{i_{\mu+1},\dots,i_{\mu-1}} \Pi^\mu_{i,i_{\mu+1},\dots,i_{\mu-1}} \prod_{\kappa\neq\mu} x^\kappa_{i_\kappa}$ into a random variable with mean zero, and standard deviation of order $1/\sqrt{N^{p-1}}$. Player μ is interested in the expected payoffs the different possible actions i in her action space return to her. These expected payoffs are given by $\sum_{i_{\mu+1},\dots,i_{\mu-1}} \Pi^\mu_{i,i_{\mu+1},\dots,i_{\mu-1}} \prod_{\kappa\neq\mu} x^\kappa_{i_\kappa}$ (for her action i), indicating that the expected payoffs for the different actions only differ by amounts proportional to $1/\sqrt{N^{p-1}}$.Thus, in order to turn these differences between the expected payoffs of different actions into appreciably different attractions, Q_i^μ, the intensity of choice β must be of order $\sqrt{N^{p-1}}$. In other words, as either N or p increase, the expected payoffs for the different actions i become less and less distinguishable (each of them is an average over an increasing number of terms). In order to exploit these differences in a meaningful way, the players have to scale up their intensity of choice β accordingly.

We take this into account by writing $\beta = N^{(p-1)/2}\tilde\beta$, where $\tilde\beta$ is independent of N and p. Introducing $Q_i^\mu = N^{-(p-1)/2}\tilde Q_i^\mu$ then leads to $\beta Q_i^\mu = \tilde\beta \tilde Q_i^\mu$, i.e., we can re-write Eq. (1) of the main paper in the following form:

$$x_i^\mu(t+1) = \frac{\exp[\tilde\beta \tilde Q_i^\mu(t)]}{\sum_k \exp[\tilde\beta \tilde Q_k^\mu(t)]}, \tag{S1}$$

where

$$\tilde Q_i^\mu(t+1) = (1-\alpha)\tilde Q_i^\mu(t) + N^{(p-1)/2} \times \sum_{i_{\mu+1},\dots,i_{\mu-1}} \Pi^\mu_{i,i_{\mu+1},\dots,i_{\mu-1}} \prod_{\kappa\neq\mu} x^\kappa_{i_\kappa}(t). \tag{S2}$$

We now further introduce $\tilde x_i^\mu = N x_i^\mu$, and $\tilde\Pi^\mu_{i,i_{\mu+1},\dots,i_{\mu-1}} = N^{-(p-1)/2}\Pi^\mu_{i,i_{\mu+1},\dots,i_{\mu-1}}$. Carrying out these transformations we arrive at

$$\begin{aligned}\tilde x_i^\mu(t+1) &= \frac{\exp[\tilde\beta \tilde Q_i^\mu(t)]}{\sum_k \exp[\tilde\beta \tilde Q_k^\mu(t)]},\\ \tilde Q_i^\mu(t+1) &= (1-\alpha)\tilde Q_i^\mu(t) + \sum_{i_{\mu+1},\dots,i_{\mu-1}} \tilde\Pi^\mu_{i,i_{\mu+1},\dots,i_{\mu-1}} \prod_{\kappa\neq\mu} \tilde x^\kappa_{i_\kappa}.\end{aligned} \tag{S3}$$

This is exactly of the same form as Eq. (1) in the main paper, but now $\tilde{\beta}$ does not scale with N or p. We also have $\sum_i \tilde{x}_i^\mu = N$, and the re-scaled payoff matrix elements $\tilde{\Pi}^\mu_{i,i_{\mu+1},\ldots,i_{\mu-1}}$ have a standard deviation of order $N^{-(p-1)/2}$. We can remove the tildes and write

$$
\begin{aligned}
x_i^\mu(t+1) &= \frac{\exp[\beta Q_i^\mu(t)]}{\sum_k \exp[\beta Q_k^\mu(t)]},\\
Q_i^\mu(t+1) &= (1-\alpha)Q_i^\mu(t) + \sum_{i_{\mu+1},\ldots,i_{\mu-1}} \Pi^\mu_{i,i_{\mu+1},\ldots,i_{\mu-1}} \prod_{\kappa\neq\mu} x^\kappa_{i_\kappa},
\end{aligned}
\tag{S4}
$$

with β order N^0 and p^0, $\sum_i x_i^\mu = N$, and

$$
\left\langle \Pi^\mu_{i_\mu,i_{\mu+1},\ldots,i_{\mu-1}} \Pi^\nu_{i_\nu,i_{\nu+1},\ldots,i_{\nu-1}} \right\rangle = \begin{cases} \frac{1}{N^{p-1}} & \mu=\nu \\ \frac{\Gamma}{(p-1)N^{p-1}} & \mu\neq\nu. \end{cases} \tag{S5}
$$

This is the starting point of the further analysis.

S2. GENERATING FUNCTIONAL ANALYSIS

Following [1, 2], we perform a generating functional analysis of the Sato-Crutchfield dynamics (i.e., the continuous limit of EWA). This will lead to an effective dynamics that is representative of the continuous limit of the EWA system, for large values of N, for typical realizations of the payoffs, and after averaging over the ensemble of random games. The fixed points of the effective dynamics are far easier to study analytically than those of the the Sato-Crutchfield equations for any particular random game.

Consider the dynamics

$$
\frac{\dot{x}_i^\mu(t)}{x_i^\mu(t)} = -r^{-1}\ln x_i^\mu(t) + \sum_{i_{\mu+1},\ldots,i_{\mu-1}} \Pi^\mu_{i,i_{\mu+1},\ldots,i_{\mu-1}} \prod_{\kappa\neq\mu} x^\kappa_{i_\kappa}(t) - \rho^\mu(t) + h_i^\mu(t). \tag{S6}
$$

This is identical to the Sato-Crutchfield dynamics, Eq. (4) in the main paper, except that we have added arbitrary functions $h_i^\mu(t)$ to generate response functions—these will later be set to zero. Recall that the normalization term $\rho^\mu(t)$ is defined such that the $x_i^\mu(t)$ have mean 1.

We define a generating functional

$$
Z[\psi] = \int \mathcal{D}[x]\,\delta(\text{equations of motion}) \exp\left(\mathrm{i}\sum_{\mu,i}\int \mathrm{d}t\, x_i^\mu(t)\psi_i^\mu(t)\right), \tag{S7}
$$

where δ(equations of motion) is used to mean that the integral is performed over realizations of (S6). Writing these delta functions in Fourier form yields

$$
\begin{aligned}
Z[\psi] = \int \mathcal{D}[x,\widehat{x}]\, \exp\Bigg(\mathrm{i}\sum_{\mu,i}\int \mathrm{d}t \Bigg\{ \widehat{x}_i^\mu(t)\Bigg(\frac{\dot{x}_i^\mu(t)}{x_i^\mu(t)} + r^{-1}\ln x_i^\mu(t) - \sum_{i_{\mu+1},\ldots,i_{\mu-1}} \Pi^\mu_{i,i_{\mu+1},\ldots,i_{\mu-1}} \prod_{\kappa\neq\mu} x^\kappa_{i_\kappa}(t) + \rho^\mu(t) - h_i^\mu(t)\Bigg) \\
+ x_i^\mu(t)\psi_i^\mu(t)\Bigg\}\Bigg).
\end{aligned}
\tag{S8}
$$

The factor in this expression depending on the payoff elements is

$$
Z_\Pi = \exp\left(-\mathrm{i}\sum_{\mu,i_1,\ldots,i_p}\int \mathrm{d}t\, \Pi^\mu_{i_\mu,i_{\mu+1},\ldots,i_{\mu-1}} \widehat{x}^\mu_{i_\mu}(t) \prod_{\kappa\neq\mu} x^\kappa_{i_\kappa}(t)\right). \tag{S9}
$$

Averaging this over the payoff elements gives

$$
\begin{aligned}
\overline{Z_\Pi} = \prod_{i_1,\ldots,i_p} \exp\Bigg\{ -\frac{1}{2N^{p-1}} \sum_\mu \int \mathrm{d}t \int \mathrm{d}t' \Bigg[\widehat{x}^\mu_{i_\mu}(t)\widehat{x}^\mu_{i_\mu}(t') \Bigg(\prod_{\kappa\neq\mu} x^\kappa_{i_\kappa}(t)\Bigg)\Bigg(\prod_{\lambda\neq\mu} x^\lambda_{i_\lambda}(t')\Bigg) \\
+\Gamma \sum_{\nu\neq\mu} \widehat{x}^\mu_{i_\mu}(t)\widehat{x}^\nu_{i_\nu}(t')\Bigg(\prod_{\kappa\neq\mu} x^\kappa_{i_\kappa}(t)\Bigg)\Bigg(\prod_{\lambda\neq\nu} x^\lambda_{i_\lambda}(t')\Bigg)\Bigg]\Bigg\},
\end{aligned}
\tag{S10}
$$

which can be written as

$$\overline{Z_\Pi} = \exp\Bigg\{ -\frac{N}{2}\int \mathrm{d}t \int \mathrm{d}t' \sum_\mu \Bigg(L^\mu(t,t') \prod_{\kappa\neq\mu} C^\kappa(t,t') + \Gamma \sum_{\nu\neq\mu} K^\mu(t,t')K^\nu(t',t) \prod_{\kappa\notin\{\mu,\nu\}} C^\kappa(t,t')\Bigg)\Bigg\}, \tag{S11}$$

where we have introduced the functions

$$\begin{aligned} C^\mu(t,t') &= \frac{1}{N}\sum_i x_i^\mu(t)x_i^\mu(t'), \\ K^\mu(t,t') &= \frac{1}{N}\sum_i x_i^\mu(t)\widehat{x}_i^\mu(t'), \\ L^\mu(t,t') &= \frac{1}{N}\sum_i \widehat{x}_i^\mu(t)\widehat{x}_i^\mu(t'). \end{aligned} \tag{S12}$$

We can use the expression (S11) in (S8), introducing the functions C^μ, K^μ, and L^μ into the integral using delta functions, for example

$$\begin{aligned} 1 &= \int \mathcal{D}[C^\mu] \prod_{t,t'} \delta\left(C^\mu(t,t') - \frac{1}{N}\sum_i x_i^\mu(t)x_i^\mu(t')\right) \\ &= \int \mathcal{D}[C^\mu, \widehat{C}^\mu] \exp\left(\mathrm{i}N \int \mathrm{d}t \int \mathrm{d}t'\, \widehat{C}^\mu(t,t') \left(C^\mu(t,t') - \frac{1}{N}\sum_i x_i^\mu(t)x_i^\mu(t')\right)\right). \end{aligned} \tag{S13}$$

The generating functional becomes

$$\overline{Z[\psi]} = \int \mathcal{D}[C,\widehat{C},K,\widehat{K},L,\widehat{L}]\, \exp(N(\psi + \Phi + \Omega)), \tag{S14}$$

where

$$\Psi = \mathrm{i}\sum_\mu \int \mathrm{d}t \int \mathrm{d}t' \left(\widehat{C}^\mu(t,t')C^\mu(t,t') + \widehat{K}^\mu(t,t')K^\mu(t,t') + \widehat{L}^\mu(t,t')L^\mu(t,t')\right) \tag{S15}$$

results from the introduction of C, K, and L into the integral,

$$\Phi = -\frac{1}{2}\sum_\mu \int \mathrm{d}t \int \mathrm{d}t' \left(L^\mu(t,t') \prod_{\kappa\neq\mu} C^\kappa(t,t') + \Gamma \sum_{\nu\neq\mu} K^\mu(t,t')K^\nu(t',t) \prod_{\kappa\notin\{\mu,\nu\}} C^\kappa(t,t')\right) \tag{S16}$$

results from the average over the payoff elements, and

$$\begin{aligned} \Omega = \frac{1}{N}\sum_{\mu,i} \ln\Bigg\{ &\int \mathcal{D}[x_i^\mu, \widehat{x}_i^\mu]\, p_{i,0}^\mu(x_i^\mu(0)) \exp\left(\mathrm{i}\int \mathrm{d}t\, x_i^\mu(t)\psi_i^\mu(t)\right) \exp\left(\mathrm{i}\int \mathrm{d}t\, \widehat{x}_i^\mu(t) \left(\frac{\dot{x}_i^\mu(t)}{x_i^\mu(t)} + \frac{1}{r}\ln x_i^\mu(t) + \rho^\mu(t) - h_i^\mu(t)\right)\right) \\ &\times \exp\left[-\mathrm{i}\int \mathrm{d}t \int \mathrm{d}t' \left(\widehat{C}^\mu(t,t')x_i^\mu(t)x_i^\mu(t') + \widehat{K}^\mu(t,t')x_i^\mu(t)\widehat{x}_i^\mu(t') + \widehat{L}^\mu(t,t')\widehat{x}_i^\mu(t)\widehat{x}_i^\mu(t')\right)\right]\Bigg\} \end{aligned} \tag{S17}$$

contains the integral over x and $\widehat{x}$. Here, $p_{i,0}^\mu(\cdot)$ represents the initial distribution of x_i^μ.

In the limit as $N \to \infty$, the integrals in (S14) can be performed using the saddle-point method. Extremising the exponent with respect to C^μ, K^μ, and L^μ gives the relations

$$\begin{aligned} \mathrm{i}\widehat{C}^\mu(t,t') &= \frac{1}{2}\sum_{\nu\neq\mu} \left(L^\nu(t,t') \prod_{\kappa\notin\{\mu,\nu\}} C^\kappa(t,t') + \Gamma \sum_{\kappa\notin\{\mu,\nu\}} K^\nu(t,t')K^\kappa(t',t) \prod_{\lambda\notin\{\mu,\nu,\kappa\}} C^\lambda(t,t')\right), \\ \mathrm{i}\widehat{K}^\mu(t,t') &= \Gamma \sum_{\nu\neq\mu} K^\nu(t,t') \prod_{\kappa\notin\{\mu,\nu\}} C^\kappa(t,t'), \\ \mathrm{i}\widehat{L}^\mu(t,t') &= \frac{1}{2}\prod_{\kappa\neq\mu} C^\kappa(t,t'), \end{aligned} \tag{S18}$$

while extremisation with respect to $\widehat{C}^\mu$, $\widehat{K}^\mu$, and $\widehat{L}^\mu$ leads to

$$
\begin{aligned}
C^\mu(t,t') &= \lim_{N\to\infty} \frac{1}{N} \sum_i \langle x_i^\mu(t) x_i^\mu(t')\rangle_\Omega \,, \\
K^\mu(t,t') &= \lim_{N\to\infty} \frac{1}{N} \sum_i \langle x_i^\mu(t) \widehat{x}_i^\mu(t')\rangle_\Omega \,, \\
L^\mu(t,t') &= \lim_{N\to\infty} \frac{1}{N} \sum_i \langle \widehat{x}_i^\mu(t) \widehat{x}_i^\mu(t')\rangle_\Omega \,,
\end{aligned} \tag{S19}
$$

where $\langle\cdot\rangle_\Omega$ represents a mean taken against a measure defined by Ω, see for example the Supplemental Material of [2] for details in a similar calculation for $p = 2$.

It can also be seen, from the definition of the generating functional, that we have

$$
\begin{aligned}
C^\mu(t,t') &= -\lim_{N\to\infty} \frac{1}{N} \sum_i \left. \frac{\delta^2 \overline{Z[\psi]}}{\delta\psi_i^\mu(t)\delta\psi_i^\mu(t')} \right|_{\psi=h=0} , \\
K^\mu(t,t') &= -\lim_{N\to\infty} \frac{1}{N} \sum_i \left. \frac{\delta^2 \overline{Z[\psi]}}{\delta\psi_i^\mu(t)\delta h_i^\mu(t')} \right|_{\psi=h=0} , \\
L^\mu(t,t') &= -\lim_{N\to\infty} \frac{1}{N} \sum_i \left. \frac{\delta^2 \overline{Z[\psi]}}{\delta h_i^\mu(t)\delta h_i^\mu(t')} \right|_{\psi=h=0} .
\end{aligned} \tag{S20}
$$

Because of normalization, $Z[\psi = 0] = 1$ for all h, so $L^\mu(t,t') = 0 \ \forall t, t'$. Due to causality, we have $K^\mu(t,t') = 0$ for $t' > t$, so that $K^\mu(t,t')K^\nu(t',t) = 0$.

This leaves $\Psi + \Phi = 0$, and if we set $\psi = 0$, and assume identical perturbations $h_i^\mu(t) = h(t)$ and initial distributions $p_{i,0}^\mu(x) = p_0(x)$ for all players and strategy components, then we have

$$
\begin{aligned}
\Omega = p \ln \Bigg\{ \int \mathcal{D}[x,\widehat{x}]\, p_0(x(0)) \exp\left(\mathrm{i} \int \mathrm{d}t\, \widehat{x}(t) \left(\frac{\dot{x}(t)}{x(t)} + \frac{1}{r} \ln x(t) + \rho(t) - h(t) \right)\right) \\
\times \exp\left[- \int \mathrm{d}t \int \mathrm{d}t' \left(\Gamma(p-1) K(t,t') C(t,t')^{p-2} x(t)\widehat{x}(t') + \frac{1}{2} C(t,t')^{p-1} \widehat{x}(t)\widehat{x}(t') \right)\right] \Bigg\}
\end{aligned} \tag{S21}
$$

where we have dropped the distinction between different players and strategy components. Each degree of freedom then has an effective generating functional

$$
\begin{aligned}
Z_{\mathrm{eff}} = \int \mathcal{D}[x,\widehat{x}]\, p_0(x(0)) \exp\left(\mathrm{i} \int \mathrm{d}t\, \widehat{x}(t) \left(\frac{\dot{x}(t)}{x(t)} + \frac{1}{r} \ln x(t) + \rho(t) - h(t) \right)\right) \\
\times \exp\left[- \int \mathrm{d}t \int \mathrm{d}t' \left(\Gamma K(t,t') C(t,t')^{p-2} x(t)\widehat{x}(t') + \frac{1}{2} C(t,t')^{p-1} \widehat{x}(t)\widehat{x}(t') \right)\right].
\end{aligned} \tag{S22}
$$

Defining $G(t,t') = -\mathrm{i}K(t,t')$, we have

$$
\begin{aligned}
Z_{\mathrm{eff}} = \int \mathcal{D}[x,\widehat{x}]\, p_0(x(0)) \exp\left(\mathrm{i} \int \mathrm{d}t\, \widehat{x}(t) \left(\frac{\dot{x}(t)}{x(t)} + \frac{1}{r} \ln x(t) + \rho(t) - h(t) \right)\right) \\
\times \exp\left[- \int \mathrm{d}t \int \mathrm{d}t' \left(\mathrm{i}\Gamma G(t,t') C(t,t')^{p-2} x(t)\widehat{x}(t') + \frac{1}{2} C(t,t')^{p-1} \widehat{x}(t)\widehat{x}(t') \right)\right],
\end{aligned} \tag{S23}
$$

which is identical to the generating functional of the effective dynamics

$$
\frac{\dot{x}(t)}{x(t)} = \Gamma \int \mathrm{d}t'\, G(t,t') C(t,t')^{p-2} x(t') - \frac{1}{r} \ln x(t) - \rho(t) + \eta(t) + h(t), \tag{S24}
$$

where $\eta(t)$ is a Gaussian random variable satisfying $\langle\eta(t)\eta(t')\rangle_* = C(t,t')^{p-1}$ and $\langle\eta(t)\rangle_* = 1$, and the functions G and C are determined by

$$\begin{aligned} G(t,t') &= \left\langle \frac{\delta x(t)}{\delta h(t')} \right\rangle_* , \\ C(t,t') &= \langle x(t)x(t')\rangle_* , \end{aligned} \tag{S25}$$

with $\langle\cdot\rangle_*$ used to denote an average over the effective dynamics (S24). Finally setting h to zero, the effective system is

$$\frac{\dot{x}(t)}{x(t)} = \Gamma \int \mathrm{d}t'\, G(t,t')C(t,t')^{p-2}x(t') - \frac{1}{r}\ln x(t) - \rho(t) + \eta(t). \tag{S26}$$

with G, C, and η defined as above.

S3. ONSET OF INSTABILITY IN THE LARGE-p LIMIT

Writing $n = p-1$ for convenience, the boundary of the stable region is given by the solution of the following equations:

$$\begin{aligned} \frac{1}{r}\ln x - \Gamma q^{n-1}\chi x - q^{n/2}z + \rho &= 0, \\ \int_{-\infty}^{\infty} \mathrm{D}z\, \frac{\partial x}{\partial z} &= q^{n/2}\chi, \\ \int_{-\infty}^{\infty} \mathrm{D}z\, x^2 &= q \\ \int_{-\infty}^{\infty} \mathrm{D}z\, x &= 1 \\ \int_{-\infty}^{\infty} \mathrm{D}z \left(\frac{\partial x}{\partial z}\right)^2 &= \frac{q}{n}, \end{aligned} \tag{S27}$$

where $\mathrm{D}z$ is a shorthand for the standard Gaussian measure $\mathrm{D}z = \frac{\mathrm{d}z}{\sqrt{2\pi}}\mathrm{e}^{-z^2/2}$.
For $\Gamma = 0$ the order parameters at the boundary of the stable region are given by Eq. (24) in the main paper. As an ansatz for the region with $\Gamma < 0$ we assume that the order parameters and the value of r on the phase boundary scale with n in the same way as they do for $\Gamma = 0$. We can write

$$\begin{aligned} q &= 1 + n^{-1}q', \\ \chi &= n^{-\frac{1}{2}}\chi', \\ r &= n^{-\frac{1}{2}}r', \\ \rho &= n^{-\frac{1}{2}}\rho', \end{aligned} \tag{S28}$$

where all primed variables are of order $\mathcal{O}(n^0)$.
If we also write $x = 1 + n^{-\frac{1}{2}}x'$, and retain only leading-order terms in q', χ', r', and ρ' t we obtain from Eq. (S27):

$$\begin{aligned} \frac{1}{r'}\ln\left(1+n^{-\frac{1}{2}}x'\right) - n^{-\frac{1}{2}}(1+q'/2)z + n^{-1}\rho' & \\ -n^{-1}\Gamma(1+q')\chi' - n^{-\frac{3}{2}}\Gamma(1+q')\chi' x' &= 0, \\ \int_{-\infty}^{\infty} \mathrm{D}z\, \frac{\partial x'}{\partial z} &= \left(1+\frac{q'}{2}\right)\chi', \\ \int_{-\infty}^{\infty} \mathrm{D}z\, x'^2 &= q', \\ \int_{-\infty}^{\infty} \mathrm{D}z\, x' &= 0, \\ \int_{-\infty}^{\infty} \mathrm{D}z \left(\frac{\partial x'}{\partial z}\right)^2 &= 1 + \frac{q'}{n}. \end{aligned} \tag{S29}$$

The linear term in x' in the first of these equations is dominated by the log term except at large x. Specifically, by using the approximation $W_{-1}(y) \approx \ln(-y)$ as $y \to \infty$, it can be seen that the linear term reaches the size of the log term when the value of x', to leading order, is

$$x' \approx x_l = \frac{n^{\frac{3}{2}} \ln n}{-\Gamma r'(1+q')\chi'}, \tag{S30}$$

while to leading order z is

$$z \approx z_l = \frac{n^{\frac{1}{2}} \ln n}{r' \left(1 + \frac{q'}{2}\right)} \tag{S31}$$

It remains only to show that the region of the real line beyond z_l makes a vanishing contribution to the integrals in Eqs. (S29). By ignoring the linear term for $z < z_l$, and the log term for $z > z_l$, the integrals over these two regions can be approximated analytically. In each case, the $z > z_l$ contribution shrinks more quickly as n grows.
Neglecting the linear term in the first relation in Eq. (S29) altogether is equivalent to making the approximation $x = 1$ in the linear term in the first equation of (S27). This yields a system of the same form as the $\Gamma = 0$ special case, except for an additional constant term, which can be solved exactly in the same manner. So, to leading order, the parameters r, χ, and q take constant values along the stability curve for large p, while ρ takes the value

$$\rho = \left(\frac{1}{2} + \Gamma\right)\sqrt{\frac{\mathrm{e}}{n}}. \tag{S32}$$

This value for ρ scales with n in the same way as it does in our ansatz, so the ansatz is indeed valid for all negative values of Γ. This demonstrates that $r = \sqrt{\mathrm{e}(p-1)}$ is a solution of the equations for the onset of instability in the limit of large p.

S4. FURTHER NUMERICAL RESULTS: LIMIT CYCLES AND MULTIPLICITY OF FIXED POINTS

S4.1. Competitive games ($\Gamma < 0$)

In Fig. S1 we show the likelihood of converging to a limit cycle for games with negatively correlated payoff matrix elements, i.e. games in which players compete agains each other ($\Gamma < 0$). For intermediate values of α, just smaller than those for which stable fixed points are ubiquitous, limit cycles are seen very commonly. However, at small values of α, fixed points or limit cycles are achieved only rarely—chaos is the norm.

S4.2. Positively correlated payoffs ($\Gamma > 0$)

For positive values of the competition parameter, chaotic dynamics is rarely observed (though chaotic-appearing transients are frequently seen). Instead, for smaller values of α and Γ, limit cycles are very common, as shown in Fig. S2. In the rest of this region, EWA consistently converges to a fixed point. However, for small values of α and large values of Γ, there are many distinct fixed points that the dynamics can converge to for a given payoff matrix. This is shown in Fig. S3.

[1] M. Opper, S. Diederich, Phys. Rev. Lett. **69** 1616-1619 (1992)
[2] T. Galla, J. D. Farmer, Proc. Nat. Acad. Sci (USA) **110** 1232 (2013)

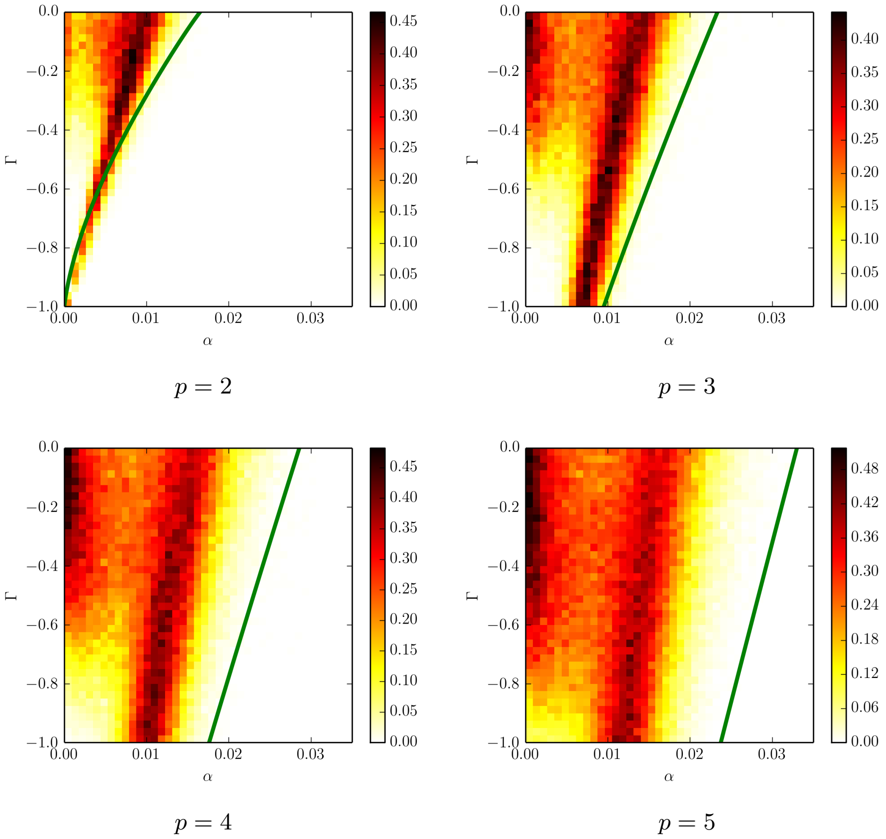

FIG. S1. Heat maps showing the fraction of 500 random initial conditions for which the EWA system converged to a limit cycle according to the heuristic in the main paper, for negative Γ. Limit cycles appear in a narrow band at intermediate values of α. The green curves show the boundaries of the stable region as derived in the main paper (Eq. (21)).

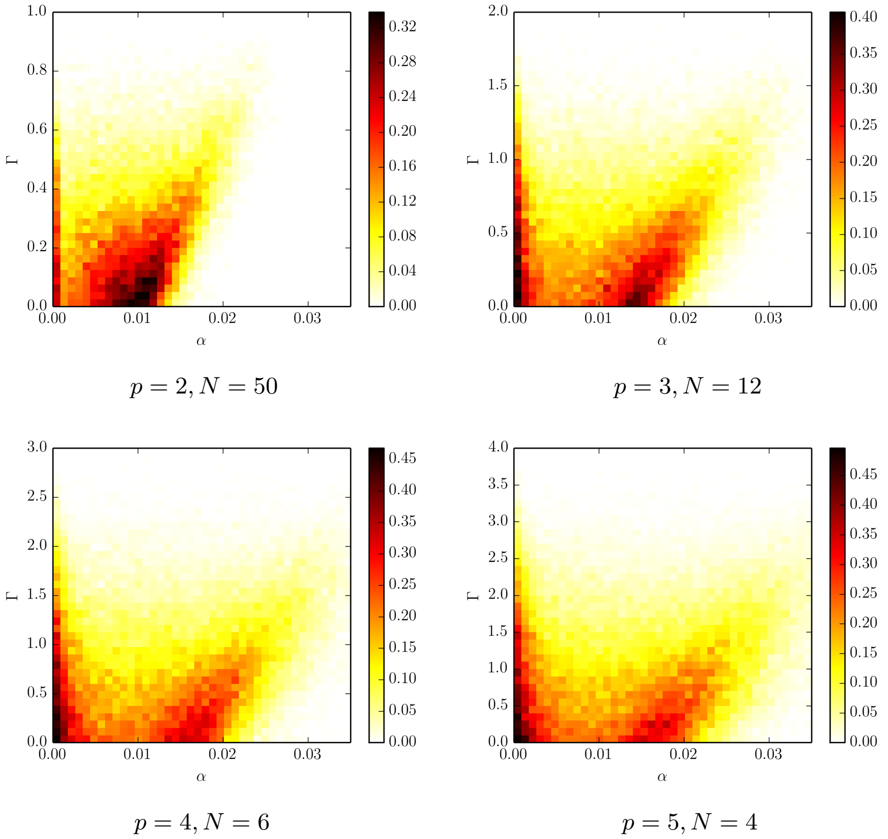

FIG. S2. Heat maps showing the fraction of 500 random initial conditions for which the EWA system converged to a limit cycle according to the heuristic in the main paper, for positive Γ. Limit cycles appear most commonly when the payoffs are weakly correlated.

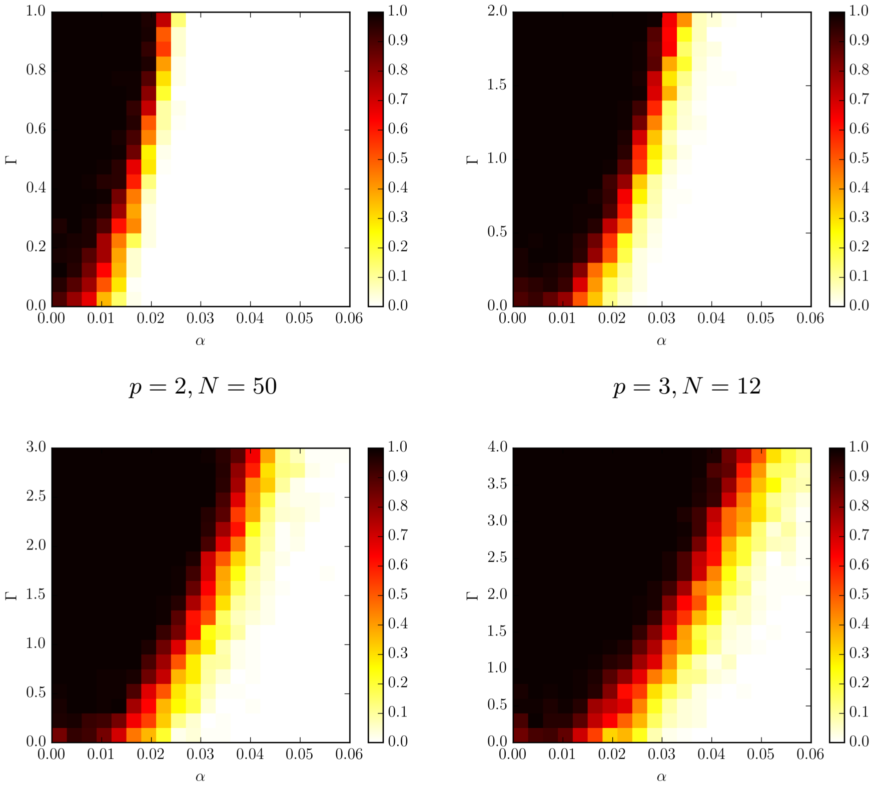

$p = 2, N = 50$ $p = 3, N = 12$

$p = 4, N = 6$ $p = 5, N = 4$

FIG. S3. Heat maps showing the fraction of 20 independent payoff matrices for which the EWA dynamics converged to multiple distinct fixed points for different initial conditions. For each payoff matrix, the EWA system was iterated for 100 different initial conditions, with fixed points being detected as explained in the main paper. Fixed points were considered to be identical if the relative distance between each component was less than 0.1.

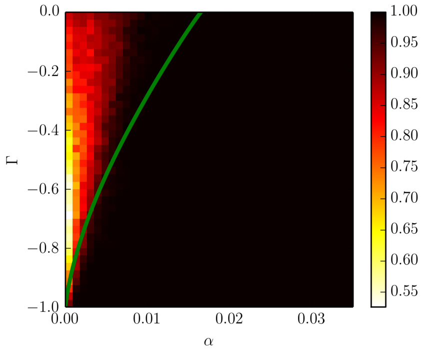

(a) $p = 2, N = 10$.

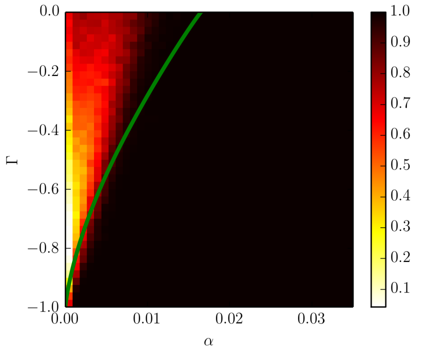

(b) $p = 2, N = 20$.

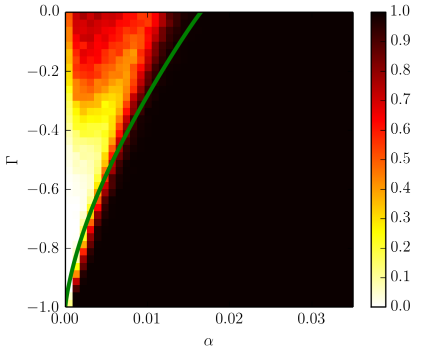

$p = 2, N = 50$

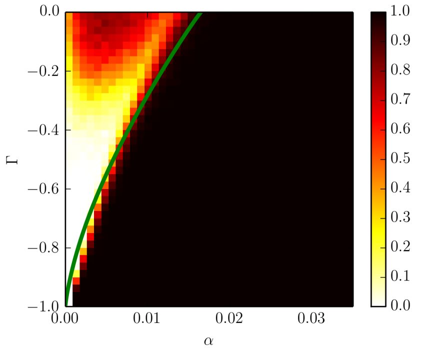

$p = 2, N = 100$

FIG. S4. Heat maps showing the dependence on N of the stable region for $p = 2$ and $\Gamma < 0$. For each set of parameters the system was iterated for 500 random initial conditions. The heat maps show the fraction that converged to a fixed point (numerical convergence criteria are described in the main paper). The size of the unstable region grows with the size N of the payoff matrix, but begins to converge around $N = 50$. The green curves are the stability curves as derived from the generating functional analysis in the main paper.
